# Reconstructing Historical Land Use and Anthropogenic Inputs in Lake Victoria Basin: Insights from PAH and n-Alkane Trends

**DOI:** 10.3390/toxics13020130

**Published:** 2025-02-10

**Authors:** Camille Joy Enalbes, Dennis M. Njagi, Chen Luo, Daniel Olago, Joyanto Routh

**Affiliations:** 1 Department of Thematic Studies—Environmental Change, Linköping University, 581 83 Linköping, Sweden; cjenalbes@gmail.com (C.J.E.); dennis.njagi@liu.se (D.M.N.); chen.luo@liu.se (C.L.); 2 Department of Geology, University of Nairobi, Nairobi 30197, Kenya; dolago@uonbi.ac.ke

**Keywords:** polycyclic aromatic hydrocarbons, n-alkanes, urbanization, combustion, land use, Lake Victoria basin

## Abstract

Over the past century, human activities have profoundly transformed global ecosystems. Lake Victoria in East Africa exemplifies these challenges, showcasing the interplay of anthropogenic pressures driven by land use changes, urbanization, agriculture, and industrialization. Our comprehensive study investigates polycyclic aromatic hydrocarbons (PAHs) and n-alkanes in the lake and its catchment to trace their sources and historical deposition. Sediment cores were collected from six sites within the catchment, representing diverse landforms and human activities, ensuring a comprehensive understanding of the basin. The results indicate significant spatial and temporal variations in both PAH and n-alkane profiles, reflecting diverse land use changes and development trajectories in the basin. Urban sites often exhibited higher concentrations of PAHs and short-chain n-alkanes, indicative of anthropogenic sources such as fossil fuel combustion, the input of petroleum hydrocarbons, and industrial emissions. In contrast, rural areas showed low PAH levels and a dominance of long-chain n-alkanes from terrestrial plant waxes. The n-alkane ratios, including the Carbon Preference Index and the Terrigenous–Aquatic Ratio, suggested shifts in organic matter sources over time, corresponding with land use changes and increased human activities. A mid-20th century shift toward increased anthropogenic contributions was observed across sites, coinciding with post-independence development. The mid-lake sediment core integrated inputs from multiple sub-catchments, providing a comprehensive record of basin-scale changes. These findings highlight three distinct periods of organic matter input: pre-1960s, dominated by natural and biogenic sources; 1960s–1990s, marked by increasing anthropogenic influence; and post-1990s, characterized by complex mixtures of pyrogenic, petrogenic, and biogenic sources. This study underscores the cumulative environmental and aquatic ecosystem effects of urbanization (rural vs. urban sites), industrialization, and land use changes over the past century. The combined analyses of PAHs and n-alkanes provide a comprehensive understanding of historical and ongoing environmental impacts, emphasizing the need for integrated management strategies that address pollutant inputs to preserve Lake Victoria’s ecological integrity.

## 1. Introduction

During the last century, anthropogenic activities such as urbanization, agriculture, and the overutilization of natural resources have exerted significant pressures on global ecosystems, resulting in escalating pollution levels and threats to biodiversity [1,2]. Lake Victoria in East Africa, covering approximately 68,800 km^2^, is the world’s second-largest freshwater body. It exemplifies this environmental challenge, which has been unfolding rapidly since the colonization of East Africa [3,4,5,6,7,8,9,10]. This vast water body sustains the livelihoods of nearly 40 million people across Uganda, Kenya, and Tanzania, providing essential ecological services vital for agriculture, fishing, energy, and transportation [5,11,12,13]. The urgency of the environmental challenges facing Lake Victoria is underscored by the fact that the communities that depend on it for their livelihood also contribute to its degradation by discharging untreated sewage, agricultural runoff, and industrial effluents. These pollutants drive increased productivity through algal blooms while simultaneously causing oxygen depletion and declining water quality, which, in turn, disrupt the delicate balance of the lake’s aquatic ecosystem [14,15,16].

The emergence of these environmental concerns can be traced back to the early 1900s, spurred by British colonization, the construction of the East African Railways, and the subsequent population boom, agriculture, and urban expansion [16,17]. This historical context provides a deep understanding of the environmental changes over the past century and underscores the need for comprehensive research to address these challenges. Following their independence in the 1960s, the three East African nations (Kenya, Tanzania, and Uganda) prioritized economic development, which catalyzed urban development and rapid population expansion within the lake’s catchment [5,11,12]. This unprecedented growth came at an ecological cost, marked by widespread deforestation, intensified commercial fishing, and the increased environmental discharge of household, industrial, and agricultural waste, leading to elevated nutrient levels of nitrogen and phosphorous and various organic pollutants in surface water bodies, including Lake Victoria [15,16,18]. Consequently, the shift in the lake’s trophic status caused an ecological imbalance, resulting in toxic blooms, oxygen depletion in the water column, and a detrimental impact on the fish population. This imbalance underscores the urgent need for effective management strategies, guided by in-depth investigations and practical experiences, to restore and sustain the ecological integrity of Lake Victoria [9,10,19,20,21,22].

Farming and industrial emissions are primary sources of nutrients that drive eutrophication, but black carbon (BC) from slash-and-burn agricultural practices also contributes to pollution [11,23,24,25,26,27,28]. BC, formed during the incomplete combustion of biomass and fossil fuels [23,24,29,30], is transported via air and freshwater, potentially releasing nitrogen oxides (NOxs) that trigger algal blooms. Thus, identifying BC sources is crucial for effective ecosystem management [23,24]. Since polycyclic aromatic hydrocarbons (PAHs) and n-alkanes are produced during combustion processes, they can serve as useful biomarkers for tracing the origin of BC [31,32]. PAHs are organic compounds with two or more fused benzene rings that arise from various sources, including pyrogenic and petrogenic sources. Pyrogenic PAHs are derived from the incomplete combustion of fossil fuels, wood, and biomass, resulting in high-molecular-weight compounds, usually with 4–6 benzene rings [32,33,34,35,36,37,38,39]. Petrogenic sources include crude oil seeps, refined petroleum, and petroleum products such as asphalt. They are characterized by low-molecular-weight 2–3 benzene rings and alkylated compounds, as well as biological derivatives from the early diagenesis of organic matter. While petroleum products contribute to PAHs, diagnostic ratios with defined cutoffs are essential for distinguishing between pyrogenic and petrogenic sources [37,38]. Anthropogenic activities, such as industrial emissions and urban runoff, significantly contribute to the transport and deposition of PAHs in sedimentary ecosystems [37,38]. n-Alkanes are straight-chain saturated hydrocarbons that can provide insights into productivity shifts and environmental contamination due to their stability and source-specific signatures. The carbon chain length distribution of n-alkanes can, additionally, indicate the origin of organic matter and help distinguish aquatic algae and terrestrial higher plants [39,40]. Long-chain n-alkanes are associated with waxes from higher plant leaves and grasses, reflecting terrestrial inputs. Short-chain n-alkanes are primarily derived from algae and microorganisms, indicating aquatic productivity changes [39,41]. Additionally, anthropogenic activities contribute to n-alkane inputs through petroleum spills, fossil fuel combustion, and industrial discharges, often resulting in an increased presence of medium-chain n-alkanes and a dominant even carbon number distribution [42,43,44]. By analyzing n-alkane distributions and ratios, such as the Carbon Preference Index (CPI) and the Terrigenous-to-Aquatic Ratio (TAR), we can infer past environmental conditions, the dominant vegetation cover type, and the impact of human activities on the ecosystem [40,41,45].

Lake Victoria’s ecological challenges are characteristic of broader global concerns regarding anthropogenic impacts on aquatic ecosystems from ongoing natural and anthropogenic activities within the catchment. This study addresses the deposition of PAHs and n-alkanes into Lake Victoria and traces their sources and contributions in tandem with the BC pool. Few studies have attempted to reconstruct the historical deposition patterns of these components by linking land use changes and pollution dynamics in both the catchment and the open lake on decadal scales. This study’s integrated approach, which examines the interplay between organic matter inputs and anthropogenic activities, provides a holistic understanding of the factors shaping the lake’s ecological dynamics. Moreover, simultaneously identifying the different pollutant sources and connecting them to broader environmental transformations contextualizes their impact within the larger framework of land use changes, climate variability, and human interventions. These findings are expected to provide actionable insights into effective environmental management strategies and facilitate more sustainable conservation efforts in the Lake Victoria basin region.

## 2. Materials and Methods

### 2.1. Study Area

Five riparian nations (Kenya, Tanzania, Uganda, Rwanda, and Burundi) share the Lake Victoria basin’s expansive 194,200 km^2^ catchment. The equatorial climate in the basin is influenced by the high elevation and surrounding mountains, resulting in moderate temperatures ranging from 20 to 35 °C and annual precipitation of ca. 1000 to 1500 mm. The lake is shallower (average depth of 41 m) than other East African Rift system lakes. Ongoing natural and anthropogenic processes within the catchment contribute to substantial inputs of various nutrients in the extensive lake [11,18]. The main rivers that drain the catchment in Kenya are Nzoia, Gucha-Migori, Sondu, Yala, Nyando, and Sio [11], transporting sediments and pollutants as they flow downstream from the highlands into the Lake Victoria basin.

**Figure 1:**
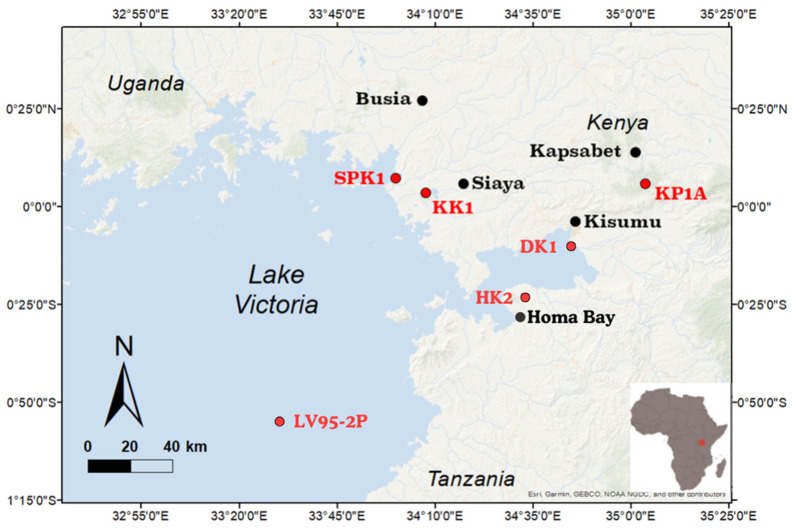
Study sites in the Lake Victoria Basin in Kenya.

For this study, six sites were selected within the Lake Victoria catchment, including the Kapsabet, Busia, Kisumu, Homa Bay, and Siaya counties and a site located in the middle of the lake (Figure 1). Siaya and Busia represent lowland areas, Kisumu and Homa Bay represent the midlands, and Kapsabet is in the highlands [46]. This selection was made to encompass diverse landforms, reflecting various habitats and human land use practices in the region. Agriculture, forestry, and fishing are significant contributors to Kenya’s GDP, with Lake Victoria playing a crucial role in contributing to the livelihoods of the local population through transportation, fisheries, and other ecosystem goods and services [6,47]. Siaya relies heavily on agriculture, with aquaculture and fishing also being significant occupations. Similarly, Busia relies on agriculture but is known more for its thriving aquaculture industry, producing approximately 160 mt of fish annually [48]. Since the colonial era, Kisumu and Homa Bay have developed into large urban hubs along the lake margins [11]. Kisumu is also known for its large inland port and trading facilities in East Africa [49]. Agriculture is the dominant economic sector in Homa Bay County, engaging approximately 74% of the county’s labor force [50]. The municipality lies at the foot of Mount Homa, providing the inhabitants with forestry and agricultural opportunities [51]. Kapsabet, situated in the highlands, depends mainly on agriculture and forestry [52]. Over time, the expansion of agriculture and urbanization has led to changes in land use practices and environmental consequences such as increased nutrient runoff, habitat degradation, and pollution of water bodies, which have adversely affected Lake Victoria’s water quality and biodiversity [49]. The slash-and-burn practice in East Africa (including the Lake Victoria catchment) is typical for land clearing and cultivation to increase soil fertility and reduce agricultural waste [53]. However, the nutrient boost that the ash provides is short-lived and eventually followed by increased soil erosion and a decline in agricultural productivity; consequently, farmers abandon the depleted land and move to new areas, perpetuating a cycle of deforestation and environmental degradation. Moreover, BC and soot emissions from slash-and-burn practices degrade air quality and pose significant health and environmental risks [24,25].

### 2.2. Sampling

We retrieved parallel sediment cores of at least 100 cm in length from Siaya (KK1), Busia (SPK1), Homa Bay (HK2), and Kisumu (DK2) in 2015 (Table 1). A percussion corer (Atlas Copco AB, Sweden) with a PVC liner measuring 100 cm in length and 5.5 cm in diameter was used to capture the sediment cores. The peat core from Kapsabet (KP1A) was retrieved in 2014 using a Russian peat borer. The 50 cm core sections (half cores, 5 cm diameter) were stored in PVC pipes after retrieval and transported to Sweden, where they were preserved in a freezer at −20 °C until further processing. Clearance from the Ministry of Mining/Geological Survey Department, Government of Kenya, was obtained before the shipment of these cores. In 1996, a core (LV95-2P) was retrieved from Kenyan waters in the lake using a Kullenberg corer. The core is stored in the Large Lakes Observatory at the University of Minnesota—Duluth (Duluth, MN, USA), and upon request, samples were provided to us (courtesy of Professor Tom Johnson).

The cores were sliced at 1 cm intervals, freeze-dried, lightly crushed to homogenize the sediments, and stored in Ziplock plastic bags at room temperature. Previous investigations have generated data on the carbon, nitrogen, phosphorous, and BC content in these cores [11,54]. Sample selection for PAH and n-alkane analyses was guided based on the organic carbon content and historical record (e.g., age and land use information, if available) of the core [11]. While ^14^C dating was performed for the KP1A core (for reconstructing a long-term millennial-scale paleoclimate record [55]), ^210^Pb dating was conducted in the other sediment cores to reconstruct centennial-scale changes [11,55]. The ^210^Pb dating for Homa Bay (HK2) was not included as the sampling site was disturbed by recent agricultural activities, which mixed the ^210^Pb signals. These disturbances led to an uneven distribution of ^210^Pb in the sediment profiles, making it unreliable for reconstructing age models at these two sites. As a result, chronological interpretations for HK2 were based on stratigraphic correlations and historical records of land use changes in the region. Sediment chronology using the ^210^Pb method in LV95-2P was based on an earlier study [49].

**Table 1:** Background information on sampling sites in the Lake Victoria basin, Kenya.

Core	GPS Location	Depth and Sampling	Site Description	Altitude (m Above Sea Level)	Rainfall	Temp (°C)	Population Density
KP1A	0°05′26.0″ N, 35°09′07.0″ E	400 cm, August 2014 (17 samples)	Kapsabet lies in the Kingwal Swamp, in the upper catchment of River Yala	1863 ± 3.4 m asl	1200–2000 mm/yr	16 °C to 22 °C	311 people/km^2^ [54,56]
SPK1	0°10′ N, 34°0′ E	100 cm, June 2015 (10 samples)	Busia lies in the Sio River basin	1188 m asl; 68 m above lake level	1600 mm/yr	22.5 °C	526 people/km^2^ [11,56]
DK2	0°7′ S 34°44′ E	100 cm, June 2015 (9 samples)	Kisumu lies in the Nyando River basin; the river is fed by precipitation from the Kericho Highlands	1197 m asl, 63 m above lake level	1250 mm/yr	23 °C	554 people/km^2^ [56]
HK2	0°7′ S 34°26′ E	100 cm, June 2015 (11 samples)	Homa Bay is on the south shore of Winam Gulf	1202 m asl; 68 m above lake level	1600 mm/yr	22.5 °C	359 people/km^2^ [51,56]
KK1	0°4′ N, 34°8′ E	100 cm, June 2015 (10 samples)	Siaya lies in the Nzoia River basin	1152 m asl; 18 m above lake level	2150 mm/yr	21 °C	393 people/km^2^ [11,56]
LV95-2P	0°58′ S, 33°27′ E	100 cm, April 1995 (13 samples)	This site, lying in the middle of Lake Victoria, Tanzania, is an oxygenated and stratified water column	1133 m asl; depth—67 m	1200 mm/year	20 °C to 25 °C	[55]

### 2.3. Organic Matter Extraction and Analyses

Biomarker Extraction: The Accelerated Solvent Extractor (Thermo ASE350) was used to extract organic matter from homogenized freeze-dried sediments at high temperature and pressure to ensure efficient extraction [57]. Crushed and homogenized freeze-dried samples were weighed and placed into stainless steel cells. The quantity varied between 2 and 5 g depending on the total organic carbon (TOC) in the samples. The sediments were injected with deuterated PAH (10 µL of 100 mg/L d-anthracene and d-acenaphthene; Sigma Aldrich) and n-alkane (10 µL of 50 mg/L d-triacontane; Sigma Aldrich) to assess recovery. The samples were extracted for three cycles at 1000 psi and 100 °C. The total lipid extract was evaporated to 1.5 to 2 mL using the Buchi Syncore device at 50 °C. The extracts were then reduced to near dryness under nitrogen gas, redissolved in 300 µL of a ratio of 2:1 (dichloromethane and isopropanol; DCM: IPA), and transferred to aminopropyl cartridges for solid-phase extraction. The cartridges were eluted using 5 mL of a ratio of 2:1 of DCM: IPA. These were then evaporated under nitrogen until reduced to nearly 1.5 mL of a neutral fraction and transferred to 4 mL vials. The neutral fractions were reduced to near dryness under nitrogen and redissolved in 300 µL of hexane. The extracts were transferred to Interchim^®^ SPE6.25ws bilayer cartridges and eluted with 5 mL of hexane to obtain the n-alkane fraction. After evaporation, the solution was reduced to 1.5 mL and transferred into 4 mL vials. The cartridge was further eluted using 5 mL of 1:1 hexane/DCM to obtain the PAH fraction. All extracts were gently evaporated to near dryness under a steady stream of nitrogen and then redissolved in a fixed volume of solvent for further analyses.

Gas Chromatography–Mass Spectrometry Analyses: The alkane and PAH fractions were injected into a gas chromatograph–mass spectrometer (Agilent 7890). The gas chromatograph was interfaced with a 5975C MSD mass spectrometer (MS) with an HP-1MS (5% phenyl methyl siloxane) fused silica capillary column (30 m length x 0.25 m i.d. x 0.25 m film thickness). The PAH fraction was also taken to dryness under nitrogen and redissolved in 200 µL hexane; 45 µL of this fraction was transferred into vials. The extract was spiked with 15 µL of 2 mg/L of a deuterated PAH mix composed of deuterated naphthalene, perylene, and phenanthrene (from Cambridge Isotope Lab) as internal standards. The PAH extracts were run using the Selected Ion Mode (SIM) at 70 eV. The target compounds were the 16 PAHs. For PAH runs, the GC oven was kept at a constant temperature of 60 °C for 2 min, increased to 150 °C for 5.6 min, then to 240 °C for 14.6 min, and held isothermally at 300 °C for 29.6 min. Calibration curves (5-point for an individual PAH) were established for evaluation using the US EPA 16 PAH standard (LGC Standard). Procedural blanks and replicate analyses were performed to assess analytical precision and accuracy. The quantification and detection limits (LOD and LOQ, respectively) were calculated based on running this standard (see Supplementary Materials). The recovery of PAHs was assessed using the same standard analyzed as a sample within each extraction batch. The recovery rates for 4–6-ring PAHs were robust, achieving 100 ± 20%, while the 2–3-ring PAHs exhibited lower recovery rates with values reaching up to 60%.

The alkane fraction was reduced to dryness, redissolved in 180 µL of hexane, transferred to vials, and spiked with 20 µL of androstane. The n-alkanes were run on full-scan mode (*m*/*z* 50–500 amu). The GC oven was kept at 35 °C for 2 min, increased to 130 °C for 6.75 min, and held isothermally at 320 °C for 44.42 min. For n-alkanes, S4066 (C_14–32_ from Chiron AB) was used to identify the retention time and fragmentation pattern, and quantification was based on the response of androstane. The recovery of d-triacontane in the samples was 101 ± 26%. The identification of PAHs and n-alkanes was based on diagnostic ion fragments and the retention time of the compound in the standards. In addition, a sediment standard Svalbard Rock from the Norway Geological Survey was extracted and analyzed. The values for C_18_-C_30_ n-alkanes indicated up to 100 ± 30% compared to the certified values. The interpretation of PAHs and n-alkanes involved using specific ratios and indices to identify their dominant sources or origins, summarized in Table 2 and Table 3.

### 2.4. Statistics

To identify the relationship between PAHs and n-alkanes, principal component analysis (PCA) was conducted using the software OriginPro 2021. The analysis identified significant score-based correlations, with higher values indicating greater significance. A threshold of ±0.5 was established based on the criterion that loadings > ±0.5 are generally considered significant in multivariate analysis, indicating that the variable strongly influences the principal component [63].

### 2.5. Limitations of the Study

Due to disturbances from agricultural activities and deforestation, the upper layers in core HK2 were disturbed, making it difficult for chronology to be established at this site. Additionally, the recovery of the LMW compounds was low, which limited the ability to calculate several diagnostic PAH ratios for a more robust source apportionment analysis of PAHs.

## 3. Results

### 3.1. PAH Concentrations

**Figure 2:**
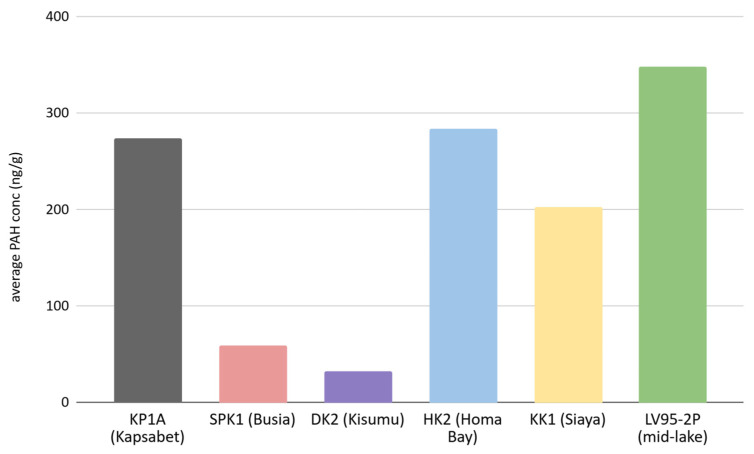
Mean PAH concentrations (ng/g dry weight) in sediment cores from sites in Lake Victoria and its catchment.

The distribution of PAHs across all study sites, ordered by abundance from highest to lowest, reveals the following sequence: naphthalene, phenanthrene, benzo[a]pyrene, benzo[*b*]fluoranthene, indeno[*1,2,3-cd*]pyrene, anthracene, fluoranthene, acenaphthylene, acenaphthene, benzo[*ghi*]perylene, pyrene, benzo[*k*]fluoranthene, dibenz[*a,h*]anthracene, chrysene, fluorene, and benz[*a*]anthracene. The concentrations (reported as ng/g dry weight) of the USEPA 16 PAHs listed as priority pollutants because of high toxicity range from 0.05 to 7011 ng/g, with several samples occurring below the detection limit of the calibration curve. The mean PAH concentration is notably highest at the LV95-2P site, with 353 ng/g. This is followed by KP1A (Kapsabet) at 270 ng/g, HK2 (Homa Bay) at 283 ng/g, KK1 (Siaya) at 200 ng/g, SPK1 (Busia) at 60 ng/g, and DK2 (Kisumu) at 31 ng/g (Figure 2).

### 3.2. PAH Ratios

While many PAH ratios have been proposed in the literature, we used those in Table 3 because their signals appear in all the sites, and it is easy to compare the different locations in the catchment. Moreover, the recovery of two- and three-ring PAHs was less efficient, which could introduce bias when relying on ratios involving these compounds for source apportionment analysis.

#### 3.2.1. ΣLow-Molecular-Weight PAH/Σ High-Molecular-Weight PAH Ratio

**Figure 3:**
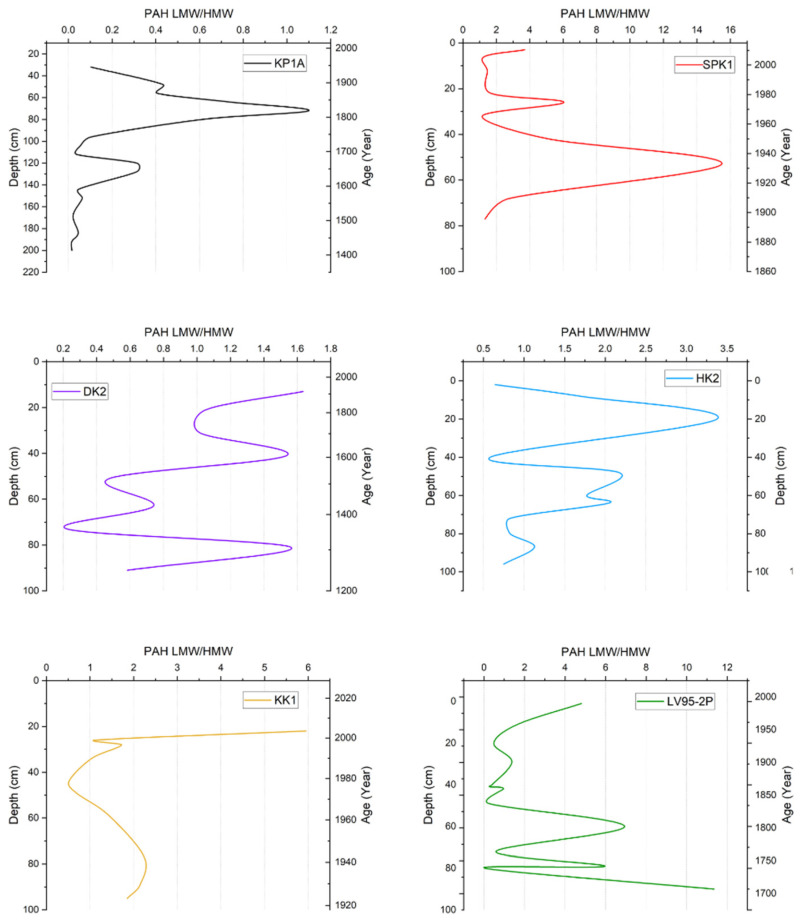
Summary of LMW/HMW PAH ratio vs. depth and age in sediment cores from Lake Victoria and its catchment.

In KP1A (Kapsabet), the LMW/HMW PAH ratio started low and remained unchanged until a spike was observed at ca. 80 cm depth before gradually declining (Figure 3). In SPK1 (Busia), the ratio increased up the core to 1 to 15 and then decreased to 1, maintaining a relatively low value for the upper part of the core. In DK2 (Kisumu), the ratio ranged from 0.46 to 1.74, with extreme variation observed throughout the core, particularly toward the bottom. In HK2 (Homa Bay), the ratio ranged from 0.51 to 3.4, with most samples ranging from 0.5 to 1.0 and a spike observed at 20 cm. In KK1 (Siaya), the values ranged from 0.5 to 6.0, with a gradual increase and then decline up the core until a sharp upward spike was observed near the surface (20 cm). In LV95-2P (Lake Victoria), the ratio ranged from 0.40 to 11.3, starting relatively high and decreasing progressively up the core; the ratio finally increased near the top of the core.

#### 3.2.2. Fluoranthene/(Fluoranthene + Pyrene) Ratio

**Figure 4:**
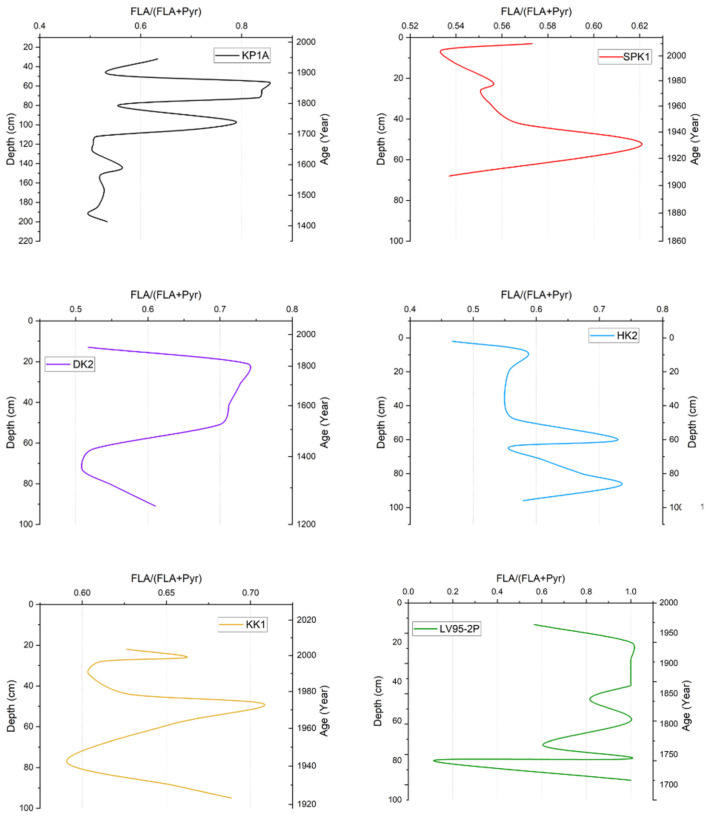
Summary of FLA/(FLA + Pyr) ratio vs. depth and age in sediment cores from sites in Lake Victoria and its catchment.

In KP1A, values were between 0.50 and 0.85 for fluoranthene/(fluoranthene + pyrene), as shown in Figure 4. The ratio was relatively low in the bottom half of the core but fluctuated between 0.50 and 0.90 for the top half of the core. In SPK1, the ratio ranged from 0.52 to 0.62. The ratio started at 0.50 at the bottom of the core before increasing to 0.62 at 50 cm. It then gradually decreased up the core to 0.52 (5 cm) and then increased slightly to 0.57 near the top. In DK2, the values ranged from 0.51 to 0.73. The ratio started at about 0.60 at the bottom and spiked between 20 and 50 cm, and then decreased to 0.51 near the top of the core. In HK2, the ratio was between 0.46 and 0.77. The ratios were mainly between 0.55 and 0.67, while two spikes occurred at 87 cm and 60 cm, respectively. A sudden decrease was observed near the top of the core at 2 cm. In KK1, the ratio ranged from 0.58 to 0.71, with substantial variability observed in the sediment layers. The LV95-2P core shows the most expansive range (0.2–1.0) in this ratio among all cores, with significant fluctuations throughout the temporal record from 1700 to 1950.

#### 3.2.3. Indeno[*1,2,3-cd*]pyrene/Indeno[*1,2,3-cd*]pyrene + Benzo[*g,h,i*]perylene Ratio

**Figure 5:**
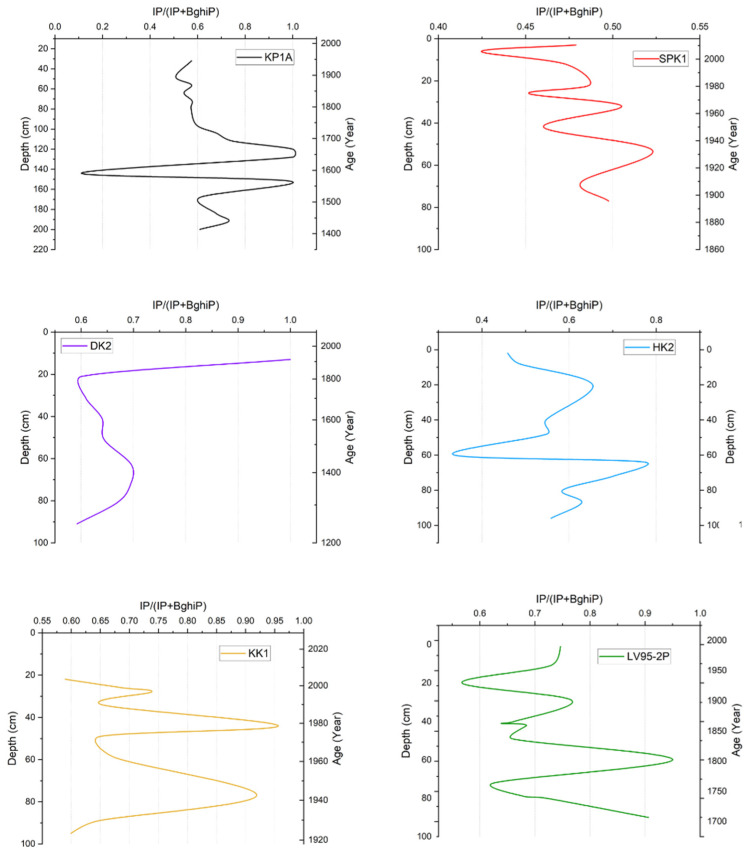
Summary of IP/(IP + BghiP) ratio vs. depth and age in sediment cores from sites in Lake Victoria and its catchment.

The IP/(IP + BghiP) ratio in KP1A was above 0.5, except for an anomalous decline to 0.10 at 140 cm (Figure 5). In SPK1, the ratio was between 0.42 and 0.52; the ratio started low and fluctuated in value up the core. In DK2, the ratio was within 0.60, which increased to 1.0 near the top of the core at 13 cm. In HK2, the ratios ranged between 0.33 and 0.77. The ratio fluctuated between 0.50 and 0.80 before it decreased sharply at 60 cm. In KK1, values ranged from 0.55 to 0.95. In LV95-2P, the ratio started at a relatively lower value at the base (0.60) and exhibited fluctuations up the core.

### 3.3. n-Alkane Concentrations

**Figure 6:**
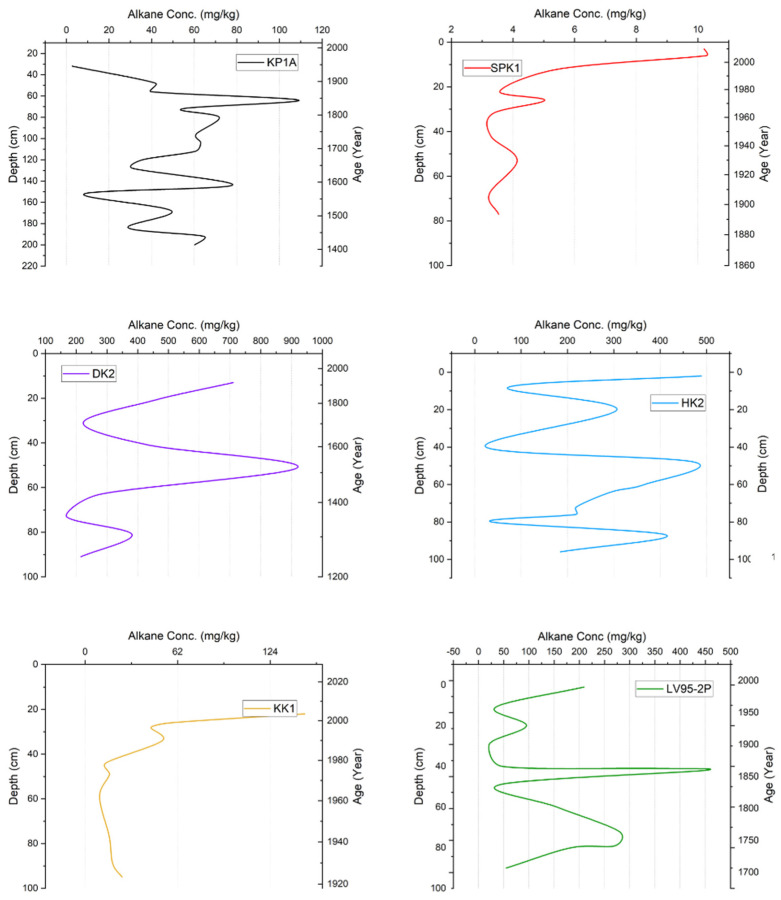
Total concentration of n-alkanes (mg/kg dry weight) in sediment cores from sites in Lake Victoria and its catchment.

The distribution of n-alkanes across all study sites (reported as mg/kg dry weight), ordered by concentration from highest to lowest, follows this sequence: The DK2 core exhibited the highest mean n-alkane concentration (n-C_11_ to n-C_33_) of 420 mg/kg compared to the other cores. At the other sites, it was 258 mg/kg (HK2), 168 mg/kg (LV95-2P), 51 mg/kg (KP1A), 40 mg/kg (KK1), and 5 mg/kg (SPK1). The average n-alkane concentration across all the sites was 157 mg/kg; the highest concentration occurred near the top of the core at most sites (Figure 6).

### 3.4. n-Alkane Ratios

#### 3.4.1. ΣLow-Molecular-Weight/ΣHigh-Molecular-Weight n-Alkane Ratio

**Figure 7:**
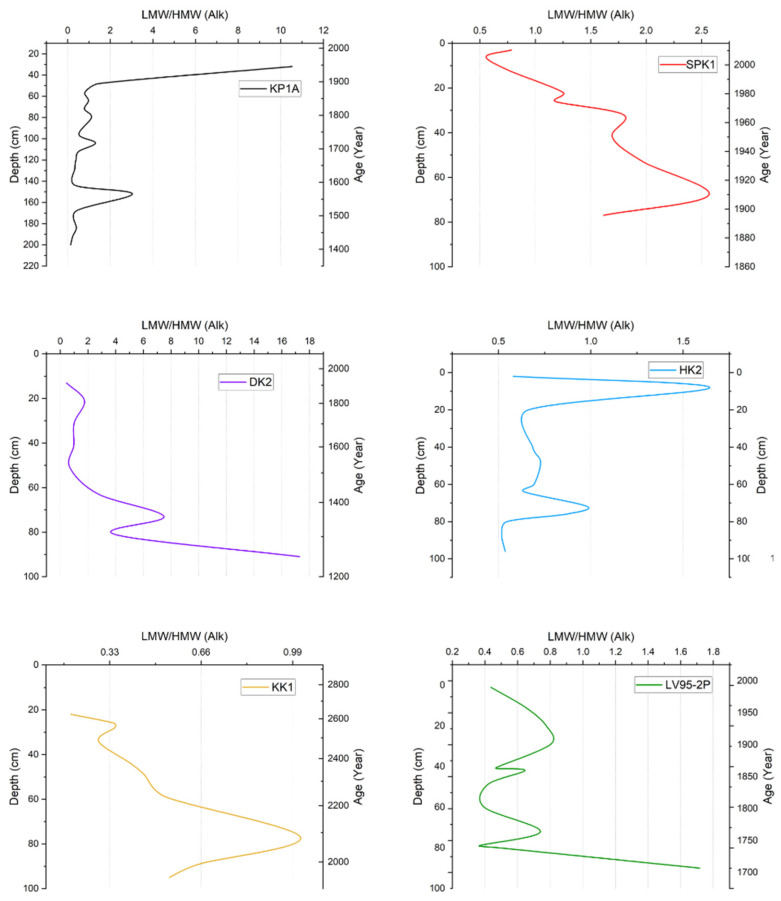
Summary of n-alkane LMW/HMW ratio vs. depth and age in sediment cores from Lake Victoria and its catchment.

In KP1A, the ratio ranged between 0 and 12.0, with most values being low except for two spikes at 160 cm and 40 cm (Figure 7). In SPK1, the LMW/HMW ratio was between 0.50 and 2.60 and decreased up the core. It started at 1.7 and then rose to 2.5 before gradually reducing up the core. In DK2, the ratio ranged from 0.46 to 17.3. The ratio decreased toward the top of the core. In HK2, the ratio ranged from 0.51 to 1.60. Most of the samples were between 0.50 and 1.0. However, spikes occurred around 70 cm and 8 cm. In KK1, the ratio was between 0.20 and 1.0. This trend was almost similar to SPK1, with the ratio decreasing near the top of the core. Values started at 0.50 and then rose to 1.0 at 70 cm before reducing up the core. Lastly, in LV95-2P, the ratio ranged from 0.40 to 1.72, starting relatively high and then decreasing erratically up the core.

#### 3.4.2. Terrigenous Aquatic Ratio

**Figure 8:**
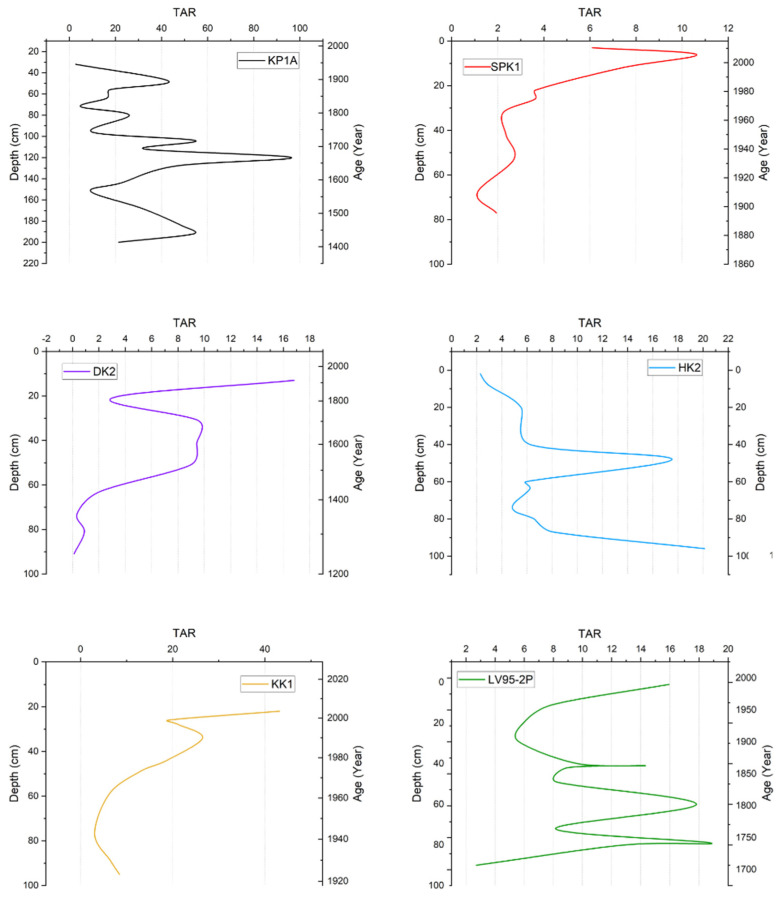
Summary of TAR vs. depth and age in sediment cores from Lake Victoria and its catchment.

TAR values in KP1A ranged from 0 to 100 and fluctuated up the core. In SPK1, the values were between 1.0 and 11.0, and the ratio generally increased up the core. In DK2, the ratios ranged from 0.34 to 16.8; its pattern was like that of SPK1 and KK1, with a spike at the top of the core and low values down the core. In HK2, the values ranged from 2.29 to 20.1. A gradual increase in values was observed downward, with two spikes at 48 cm and 96 cm. On the other hand, KK1 had a similar trend to SPK1, and values ranged from 3.0 to 50.0. In LV95-2P, values ranged from 2.0 to 20.0; the ratio started relatively low and increased up the core (Figure 8).

#### 3.4.3. Carbon Preference Index

**Figure 9:**
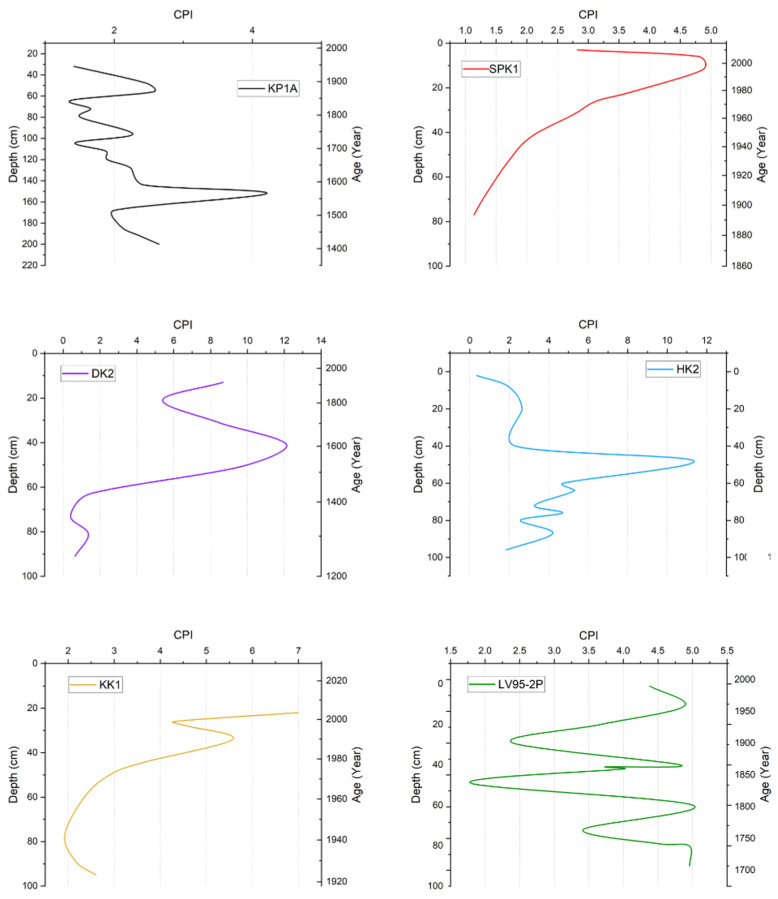
Summary of CPI ratio vs. depth and age in sediment cores from Lake Victoria and its catchment.

CPI values in KP1A were between 1.38 and 4.20; the values fluctuated up the core. In SPK1, the values were between 2.07 and 4.86 and were relatively low (1.20) at the base but increased progressively up the core, reaching a peak at 15 cm. However, the ratio then decreased to 2.75 near the top of the core. In DK2, the values were between 0.40 and 12.1. The top section had values between 5.00 and 12.0, whereas the bottom section had values between 0.40 and 1.50. In HK2, the CPI values ranged from 0.34 to 11.4. A similar trend was observed in KP1A, where the values gradually decreased up the core. However, in HK2, the ratio increased to around 48 cm. In KK1, values were between 2.00 and 7.00 and generally increased up the core. Lastly, in LV95-2P, the value fluctuated between 1.75 and 5.00 (Figure 9).

### 3.5. Statistical Trends

**Figure 10:**
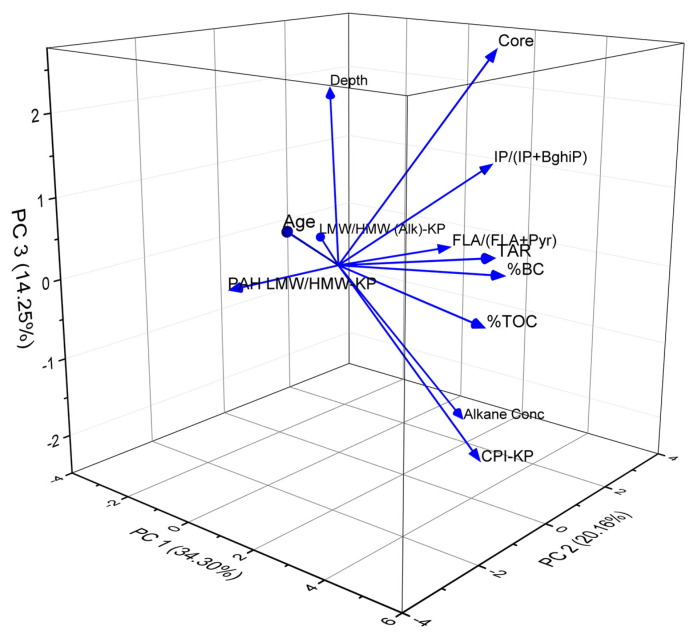
Principal component analysis showing the multivariate variation amongst different organic carbon sources (TOC, black carbon, PAH, and n-alkanes) in the Lake Victoria catchment.

The PCA results revealed complex interactions between various geochemical parameters, including the distribution pattern of pollutants and their sources, while indicating the underlying trends in the dataset (Figure 10). PCA identified three principal components (PCs) that captured the majority (68.71%) of the variance in the dataset. PC1 accounted for 34.3% of the variance and was primarily influenced by the TAR (0.45), TOC (0.41), BC (0.39), and CPI (0.34). PC2 explained 20.2% of the variance and was significantly influenced by age (−0.50), n-alkane concentration (0.41), IP/(IP + BghiP) (0.37), and depth (0.35). PC3 accounted for 14.3% of the variance and was dominated by the core location (0.54), alkane concentration (−0.49), CPI (−0.44), and depth (0.38).

**Figure 11:**
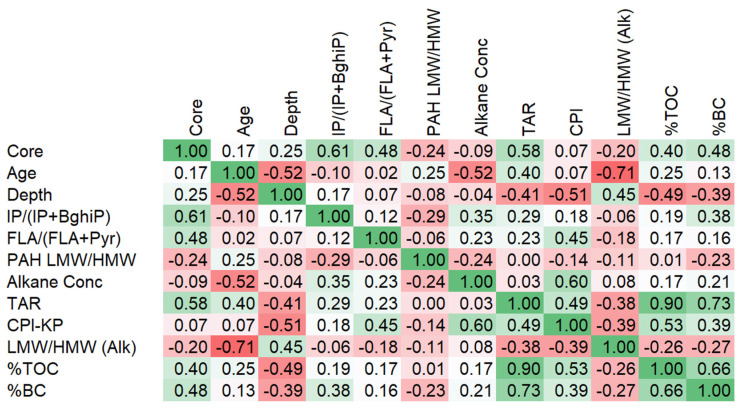
Correlation matrix of TOC, BC, PAH, and n-alkanes in the Lake Victoria catchment. Green denotes positive values, whereas red denotes negative values.

The correlation analysis of geochemical parameters in Lake Victoria basin sediments reveals several significant relationships ((*p* < 0.05); Figure 11). The TAR shows a robust correlation with %TOC (r = 0.90) and %BC (r = 0.73), indicating that terrestrial organic matter inputs are closely linked to both TOC and BC input. TOC and BC display a strong positive correlation (r = 0.66), suggesting common sources or similar preservation mechanisms. Core location correlates positively with IP/(IP + BghiP) (r = 0.61) and the TAR (r = 0.58), indicating spatial patterns in PAH sources and terrestrial inputs. The CPI shows a moderate positive correlation with alkane concentration (r = 0.60) and %TOC (r = 0.53), suggesting that higher CPI values (indicating terrestrial plant inputs) correspond with increased organic matter preservation. Age correlates negatively with the LMW/HMW alkane ratio (r = −0.71) and alkane concentration (r = −0.52), suggesting temporal changes in hydrocarbon sources and preservation. Depth correlates negatively with the CPI (r = −0.51) and %TOC (r = −0.49), indicating vertical changes in organic matter composition and preservation. FLA/(FLA + Pyr) shows a moderate positive correlation with the CPI (r = 0.45), suggesting some relationship between PAH sources and terrestrial organic matter inputs. IP/(IP + BghiP) correlates moderately with %BC (r = 0.38), indicating a link between specific PAH ratios and combustion-derived carbon.

## 4. Discussion

Land use changes in the catchment associated with agricultural practices, urbanization, and industrial development impacted the nutrient and BC flux into Lake Victoria. Population growth, surging up to 1022%, and agriculture expansion of up to 48% in some areas around the lake margin since the 1960s [11] have contributed to the lake’s trophic status. Rising BC levels have been closely linked to population growth, land use changes, and agricultural practices [11,17,64,65], while global trends in fossil fuel combustion further exacerbate BC inputs [29]. The observed increase in BC at catchment sites aligns with these trends [11]. Notably, BC sources, a key indicator of anthropogenic activities in the catchment, can be further clarified through PAH and n-alkane analyses. Their covariance and statistical correlations offer valuable insights into causality and source apportionment.

### 4.1. Statistical Relevance

Multivariate statistical approaches unraveled the complex environmental history in the LVB, reflecting the interplay of natural processes and anthropogenic influences over time [66,67]. The strong loading of TOC and BC on PC1 underscores the significance of organic carbon inputs to the lake. The positive correlation with depth suggests an increasing trend in organic matter accumulation over time, potentially linked to land use changes and increased anthropogenic activities in the catchment [7,11,61]. The contribution of the TAR to PC1 further supports this interpretation, indicating shifts in the relative inputs of terrestrial versus aquatic organic matter sources. PC2 reflects temporal and depositional patterns, as indicated by the strong negative loading of age and positive loadings of n-alkane concentration and IP/(IP + BghiP). This component suggests that younger sediments tend to have higher n-alkane concentrations and different PAH source signatures than older deposits, reflecting increasing anthropogenic inputs over time [62]. PC3 captures spatial variations in sediment composition, highlighting distinct organic matter signatures across different sampling locations in the catchment. The opposing loadings of alkane concentration and the CPI further support these contrasting characteristics and reflect various land use practices and site-specific environmental conditions that influence organic matter deposition and preservation across the basin. The PCA results suggest that terrestrial inputs and combustion sources primarily control organic matter composition. Temporal variations in sediment composition reflect growing anthropogenic influence, while spatial differences highlight variability in organic matter sources and preservation across the basin. These trends align with the historical context of increasing urbanization, industrial development, and land use changes in the Lake Victoria basin over the past century.

### 4.2. Geochemical Trends and Environmental Conditions

#### 4.2.1. Kapsabet

The biomarker profile of Kapsabet (KP1A) in the highlands of the upper catchment of the Lake Victoria basin exhibits significant variability, with spikes that potentially indicate episodic events or shifts in environmental conditions. The site’s proximity to the Kapsabet Forest, known for frequent natural forest fires [8], and the prevalence of slash-and-burn practices in the tea estates at higher elevations contribute to the elevated PAH levels detected in the core [68,69,70]. These findings align with the understanding that biomass burning is a primary source of HMW PAHs, emphasizing the impact of local activities on environmental contamination [64,71,72].

A transition is evident when examining the n-alkane distribution in Kapsabet sediments over time. Older sediments are characterized by a predominance of odd-carbon-numbered long-chain n-alkanes (C_27_–C_33_), indicative of terrestrial plant inputs. In contrast, recent sediments display a more mixed profile, suggesting contributions from both terrestrial and aquatic sources. This shift reflects changes in vegetation cover and increased anthropogenic inputs from sources like the tea factory in the region [73], as supported by the PAH ratios indicating a blend of biogenic and petrogenic sources [74]. This variability is particularly pronounced in the TAR, showcasing a complex pattern of fluctuations that likely mirror diverse input sources and dynamic environmental changes over time [75].

The higher organic carbon content at the Kapsabet site compared to other locations [11,54] likely plays a crucial role in enhancing PAH accumulation by facilitating their binding to organic matter, thereby reducing degradation and leaching rates [76]. The predominance of long-chain n-alkanes supports the idea that organic matter is well preserved due to the protective effect of plant waxes and the stable environmental conditions in the highlands. Furthermore, the highland topography enhances PAH accumulation by increasing atmospheric deposition through orographic effects. At the same time, the cooler temperatures at higher altitudes slow PAH degradation and volatilization processes, increasing their persistence in the environment [77].

#### 4.2.2. Busia

Compared to other sites around Lake Victoria, SPK1 displayed the lowest n-alkane concentrations and relatively low PAH concentrations, indicating fewer local anthropogenic inputs in Busia. This aligns with the site’s lowest BC concentration, indicating minimal contributions from high-temperature combustion sources from human activities [11]. Nevertheless, the biomarker profiles within the core reflect the gradual expansion of human activities and land use changes over time, underscoring the evolving environmental footprint of the region [78]. The low PAH levels and the higher proportion of LMW PAH in Busia suggest the predominant influence of petrogenic sources. This PAH trend aligns with the area’s rural and agricultural character, with fewer urban and industrial sources of PAHs compared to more developed areas surrounding Lake Victoria [48]. A distinct spike in PAH concentrations was observed in Busia around the 1940s, coinciding with the town’s developmental phase during the colonial era [79,80]. This period witnessed significant changes in land use, including agricultural expansion, early urbanization, and deforestation, which likely contributed to the heightened PAH inputs through biomass burning and the introduction of motorized vehicles [81].

The n-alkane distributions in Busia were dominated by long-chain n-alkanes, with a high CPI indicating substantial inputs from terrestrial higher plants [39]. This pattern reflects the area’s predominantly agricultural land use and natural vegetation/forest cover. The decreasing trend in the LMW/HMW n-alkane ratio up the core suggests a recent transition from mixed sources in earlier periods to more terrestrial sources. TAR values increased up the core, indicating rising terrestrial input over time. This trend mirrors the historical land use transformations in the region, particularly the expansion of agriculture and deforestation during the mid-20th century [79]. The high CPI and TAR values corroborate the dominance of terrestrial organic matter inputs, which is consistent with Busia’s rural character. Recent studies by Onyango et al. [7] highlight a decline in agricultural land in Busia County between 1990 and 2017, possibly due to urbanization and changes in land use policies. This shift may impact future trends in terrestrial inputs, potentially altering organic matter composition in lake sediments. An analysis of n-alkane data indicates that although Busia has experienced relatively limited industrial impact compared to the more urban sites, there is clear evidence of gradual changes in organic matter sources, driven by increasing human activities over time. This gradual change aligns with the steadily rising BC values in the region [11].

#### 4.2.3. Kisumu

As a significant urban center on the Kenyan shorelines of Lake Victoria, Kisumu’s biomarker profiles distinctly capture the imprint of industrial activities, transportation networks, and urban expansion on the lake’s ecosystem [7]. The LMW/HMW ratio for both PAHs and n-alkanes in Kisumu reveals a nuanced pattern of change over time. The higher values of the LMW/HMW ratios in earlier periods may indicate a prevalence of natural sources and traditional biomass burning. Conversely, a decline in this ratio as the core progresses into the recent/contemporary period suggests a growing contribution from high-temperature combustion processes associated with urban and industrial development [82,83]. Consistent with this, the PAH profile in Kisumu consistently shows a pyrogenic signature throughout the core, indicating the long-standing influence of combustion sources. The IP/(IP + BghiP) trend highlights the prevalence of wood, grass, or coal combustion as primary PAH sources throughout Kisumu’s history, potentially intensifying in recent periods [84]. The FLA/(FLA + Pyr) ratio displayed a significant increase around the early 20th century, which coincides with the post-independence era that has been marked by rapid urban and industrial growth. The shift in PAH ratios suggests a recent transition to more pyrogenic sources, reflecting evolving organic matter inputs as the city developed [85]. It likely reflects an upsurge in industrial activities, increased vehicular traffic, and alterations in energy consumption, for instance, from traditional biomass fuels (such as wood and charcoal) to fossil fuels like kerosene, diesel, and petrol for cooking, lighting, transportation, and industrial processes as the city expanded [9,18,86,87]. This trajectory aligns with BC trends, demonstrating increasing contributions from fossil fuel combustion over time, emphasizing Kisumu’s role as a major source of anthropogenic emissions impacting Lake Victoria’s ecosystem. Despite the urban setting, the overall low PAH levels in the core are notable (Figure 2). The site’s proximity to the Nyando River flowing nearby may contribute to the disturbance in sediments and potential loss of PAHs. This could reflect dilution effects or efficient dispersion mechanisms at play in the area [88], reducing PAH concentrations in the sediments. In contrast, the site exhibits the highest mean n-alkane concentrations. The Nyando Swamp likely accounts for elevated levels of n-alkanes, predominantly derived from swamp vegetation, besides contributions from anthropogenic inputs. The n-alkane distribution pattern is characterized by elevated concentrations of short-chain n-alkanes, suggesting significant contributions from petroleum hydrocarbons associated with urban runoff, vehicular emissions, and industrial discharges [42]. This aligns with the BC trends that highlight the growing influence of high-temperature sources [11]. The CPI values ranged widely, with lower CPI values in the upper layers of the core indicating increased fossil fuel contamination over time [39]. The decreasing trend in the LMW/HMW n-alkane ratio in the upper layers of the core further supports the increasing influence of anthropogenic sources, such as fossil fuel combustion and industrial activities [82]. The TAR values in the core show a consistent upward trend, indicating an increasing influence of land-derived organic matter inputs over time. However, the TAR values remain relatively low compared to those of rural sites, reflecting the significant input of aquatic and anthropogenic sources in an urban setting [40]. This trajectory aligns with the historical urban expansion of the city and the intensification of land use practices in the surrounding regions, including deforestation and wetland conversion for urban and agricultural purposes [7].

#### 4.2.4. Homa Bay

With a mix of agricultural and fishing activities, Homa Bay shows intermediate levels of anthropogenic input, reflecting its transitional nature between rural and urban environments. The n-alkane concentrations in Homa Bay are moderate, positioning it between the highly urban Kisumu region and the remote mid-lake sediments. This aligns with Homa Bay’s development status, suggesting a gradual rise in anthropogenic activities and urbanization over time, but not to the extent seen in Kisumu, the much larger urban center at the lake margin [7,89]. This increasing influence of combustion is also reflected in the BC trends, which are likely linked to both traditional biomass burning and increasing fossil fuel use [11]. The mid-20th century likely marked a turning point in PAH inputs, coinciding with post-independence economic development and population growth in the region. This period would have seen an increase in pyrogenic PAH inputs, reflecting the growth of urban infrastructure, transportation networks, and the development of small-scale industries in and around Homa Bay [10]. The observed increase in BC concentrations aligns with this timeline, providing additional evidence for the intensification of combustion-related activities. The LMW/HMW ratio for both PAHs and n-alkanes in Homa Bay shows a transition over time. Higher ratios during earlier periods indicate the predominance of natural sources and traditional biomass burning. In recent years, a decrease in this ratio suggests an increasing contribution from petrogenic sources, reflecting the town’s gradual urbanization and the introduction of more fossil fuel-based activities. The n-alkanes are characterized by a mix of short-chain and long-chain n-alkanes, indicating contributions from both aquatic and terrestrial sources. The fluctuations in the CPI suggest a combination of biogenic and petrogenic inputs [39]. This mixture would be consistent with Homa Bay’s evolving landscape, where traditional biomass-burning practices coexist with increasing fossil fuel use [90]. The variability in BC levels, particularly in the upper part of the core, further supports this interpretation of mixed combustion sources. The increasing trend in the TAR indicates the growing influence of terrestrial organic matter over time [40]. This is consistent with expanding agricultural activities and urban development in the area [89,91].

#### 4.2.5. Siaya

The relatively lower concentrations of both PAHs and n-alkanes in Siaya compared to more urbanized sites reflect its less industrialized character. However, the biomarker profiles still capture the gradual expansion of human activities and land use changes in the region over time. This gradual expansion was evident in the BC trends [11]. The total PAH levels and FLA/(FLA + Pyr) ratio in Siaya are lower than in urban centers like Kisumu or the lake sediment core, aligning with its primarily agricultural landscape and fewer industrial establishments [92,93,94]. The higher proportion of LMW PAHs in the region suggests a more significant influence of petrogenic sources, possibly linked to the gradual increase in fossil fuel use for transportation and small-scale industrial activities like gold mining [95] around the mid-20th century. The spike in PAH levels in the mid-20th century likely corresponds with late-colonial-era developments leading up to independence in 1963, during which significant infrastructural growth and the expansion of anthropogenic activities caused an increase in pyrogenic sources. The n-alkane concentrations in Siaya are relatively low compared to urban sites but still show an increasing trend over time. The LMW/HMW n-alkane ratio in the Siaya core suggests changes in hydrocarbon sources over time. This trend likely reflects the transition from predominantly natural inputs to increasing anthropogenic contributions as human activities expanded in the region. The dominance of long-chain n-alkanes and the CPI values indicate substantial inputs from terrestrial higher plants [39]. The increasing TAR values up the core are consistent with rising land-derived biological input over time [40]. This trend aligns with historical land use changes in the region, particularly the expansion of agriculture and gradual deforestation [7]. The BC trend further corroborates this narrative, with a steady increase reflecting the intensification of land use practices and growing anthropogenic effects in Siaya.

#### 4.2.6. Mid-Lake

The LV95-2P core revealed a complex mixture of PAH and n-alkane inputs, reflecting diverse land use practices and evolving development trajectories across the Lake Victoria basin [7,9,96]. PAH concentrations were highest in the mid-lake section, surpassing levels observed in both polluted urban sites and less impacted rural areas. This elevated concentration reflects the lake’s role as a sink for contaminants originating from multiple sub-catchments, with PAHs delivered via both atmospheric deposition and riverine transport [97]. The BC concentration in the lake showed an irregular rise up the core, further supporting the increasing and diverse influence of combustion-derived inputs over time [11]. The IP/(IP + BghiP) ratio indicates a steady decrease in PAH concentrations observed from the bottom of the core until a sharp rise around 100 cm, which coincides with post-independence economic development and population growth in the region [9,10]. The n-alkane concentrations in the mid-lake were intermediate compared to those of urban sites, further supporting the lake’s role in integrating diverse organic matter inputs from the catchment. The rise in TAR values reflects changes in land use and vegetation cover throughout the basin. It suggests that land-derived organic matter inputs have become more significant in recent decades, possibly due to intensified soil erosion and runoff resulting from agricultural practices and deforestation [91]. The concurrent increase in BC and TAR values suggests a growing contribution from both terrestrial sources and combustion processes. This trend aligns with historical land use changes in the region, particularly the expansion of agriculture and gradual deforestation in the catchment area. The LMW/HMW ratio for both PAHs and n-alkanes in the LV95-2P core showed a decreasing trend, pointing to varying contributions from terrestrial and petrogenic sources over time [35]. This shift suggests a transition from predominantly natural inputs to increasing anthropogenic contributions. The irregular BC trend also supports this transition. CPI values in the LV95-2P core fluctuated, indicating a mixture of petrogenic inputs and terrestrial plant matter throughout the lake’s history. Lower CPI values in the sediments suggest increased contributions from petroleum hydrocarbons due to anthropogenic activities, while higher CPI values point to dominant inputs from terrestrial vegetation [44]. This variability in CPI values reinforces the complex interplay of natural and anthropogenic sources contributing to the lake’s organic matter composition.

### 4.3. PAH Characteristics and Risks

**Figure 12:**
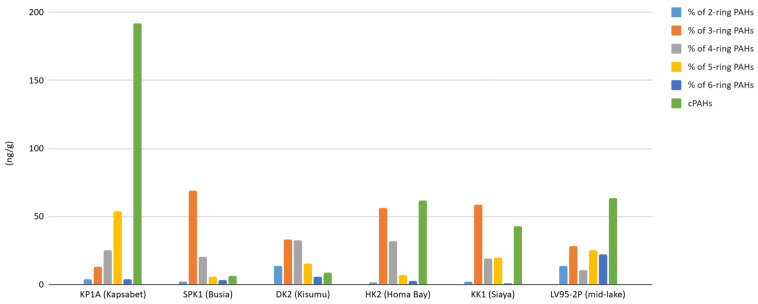
PAH ring profiles and mean carcinogenic PAH (cPAH) from sites in Lake Victoria catchment.

PAH characteristics based on ring size are useful in evaluating the sources, their behavior, and risks [98]. PAHs with two or three rings are of petrogenic origin, whereas PAHs with four to six rings are generally of pyrogenic origin [33,66,71]. This aspect is closely tied to land use and anthropogenic activities in the lake’s catchment. LMW PAHs are more mobile and susceptible to degradation or volatilization, whereas HMW PAHs survive longer in the sedimentary environment. In addition, the PAH ring profiles reveal distinct spatial patterns across the lake’s catchment (Figure 12). The mid-lake site showed the highest concentration of 2–3-ring PAHs, suggesting substantial petrogenic inputs, likely from petroleum by-products from the fishing and transport sector, atmospheric deposition, and riverine inflow from multiple sub-catchments. Urban sites like Homa Bay and Kisumu exhibit a higher proportion (compared to rural sites) of 4–6-ring PAHs, characteristic of pyrogenic sources. Busia and Siaya show lower total PAH concentrations with a balanced distribution of three-ring and four-ring PAHs, reflecting mixed inputs.

Many of the HMW PAHs are also carcinogenic, and cPAH (sum of seven potentially carcinogenic PAHs) was calculated. The cPAH concentrations vary widely across the sites, reflecting differences in anthropogenic activities and combustion intensities. Busia recorded the lowest cPAH concentrations, likely due to the site’s rural character, where agricultural activities dominate over industrial or urban influences. Siaya and Homa Bay showed a spike in cPAH during the mid-20th century, corresponding to the colonial-era developments that introduced industrialization and increased fossil fuel use. Kapsabet showed the highest variability in cPAH levels, with peak concentrations observed in the most recent sediments. This trend reflects intensified slash-and-burn practices likely associated with expanding tea estates and deforestation [54]. The elevated levels of toxic cPAHs at sites like Kapsabet underscore potential ecological and human health risks. The mid-lake core showed intermediate cPAH concentrations, highlighting its role as a sink for pollutants from diverse sources across the basin. Interestingly, Kisumu showed relatively low cPAH levels despite its urban character. This may reflect dilution effects or efficient dispersion mechanisms involving fluvial or atmospheric processes.

### 4.4. Land Use

The transformation in land use changes began during British colonization in the early 1900s, primarily driven by agricultural activities and rapid urban development, which coincided with a population increase within the lake’s catchment [11]. Forests were cleared and transformed into farmland. It was noted that a rise in agricultural production correlated with an increase in population in the Lake Victoria basin, and this trend has been connected to anthropogenic soil disturbances [11,17,99]. Rapid industrialization occurred in the region coinciding with the construction of roads and railroads, e.g., the East African Railway in Kisumu in 1901. Likewise, industries such as sugar production gained momentum after Kenya gained independence in the mid-1960s [16,54,100].

The rise in fossil fuel consumption coincided with both industrial and economic activities, resulting in the increased atmospheric deposition and surface runoff of organic matter (e.g., combustion by-products, including BC) into the catchment and lake. The distribution of PAHs and n-alkanes in the sediment cores reinforces the hypothesis that anthropogenic activities have contributed to the organic matter input, including potentially toxic components that impact human health (e.g., BC, PAHs). The increase in the concentration of these compounds coincides with BC, nutrient flux, and burgeoning development in the catchment [11,17]. Organic matter transported into the lake could have triggered the enhanced productivity observed in recent years, posing an environmental challenge [16,18,20]. Likewise, there is potential for the biomagnification of these toxic pollutants through the food chain [101], which could impact the economy of people living in the catchment and dependent on fishing as a major source of income. While PAH and n-alkane spatiotemporal trends serve as specific source indicators, and their variability reflects ongoing cultural activities, they also provide crucial insights into the environmental changes affecting Lake Victoria. These trends reveal a stark contrast between rural and urban areas, reflecting the varying influences of cultural practices and urbanization. They highlight the evolving impact of pollution sources over time, offering valuable insights to guide targeted remediation and sustainable management efforts.

### 4.5. Rural–Urban Influences and Timelines

Based on the spatial distribution and accumulation of organic matter, the environmental changes in the lake and its catchment can be summarized into two main categories reflecting processes/activities in rural and urban areas.

1. Natural/biogenic inputs of organic matter primarily influence rural areas. These regions are generally characterized by lower concentrations of PAHs and n-alkanes, a predominance of long-chain n-alkanes with high CPI values, and elevated TAR values, all indicating significant contributions from terrestrial sources. The sources include vegetation cover, agricultural practices, and the growing influence of industrialization. While organic matter inputs are primarily natural, the gradual rise in anthropogenic markers underscores the need for sustainable land use practices to protect both aquatic and terrestrial ecosystems.
2. Urban areas reflect higher anthropogenic inputs. These are characterized by higher concentrations of BC, pyrolytic PAHs, and n-alkanes. The latter demonstrates elevated levels of short-chain n-alkanes with decreasing CPI and TAR values. The inputs comprise by-products from fossil fuel combustion, industrial discharge, urban runoff, and vehicular emissions. This is also evident in the mid-lake sediment core, which reveals a distinct shift in the accumulation of these compounds, mirroring trends observed in recent sediments in the catchment. Urban development and industrial activities drive increasing pollutant loads from diverse sources, posing significant ecological risks to the lake ecosystem.

**Figure 13:**
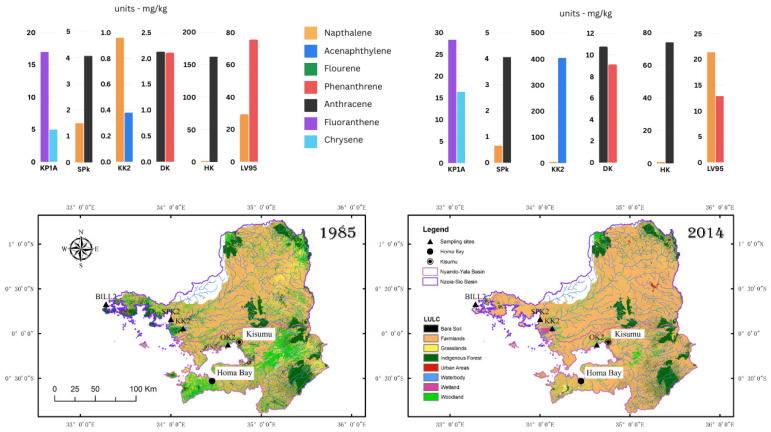
Comparison of PAH concentrations and changes in land use and land cover (LULC) in the Nyando–Yala and Nzoia–Sio basins from 1985 to 2014 (adapted from [11]). The PAH concentrations on the left are from the bottom of the core, and those on the right are from the top, showing the change in the most abundant PAH levels.

These spatial patterns are complemented by temporal trends observed across all sites. The juxtaposition of selected PAH data with land use changes provides a visual correlation between PAH levels and the alterations in land use patterns (Figure 13), underscoring the impact of anthropogenic activities on environmental pollution. The environmental changes in the lake and its catchment, based on the deposition and accumulation of organic matter, can be summarized into three main temporal periods

- Pre-1960: PAH and n-alkane profiles consistent with biomass burning and traditional agricultural practices characterize this period. The dominance of long-chain n-alkanes with high CPI values indicates substantial inputs from terrestrial vegetation. The anthropogenic influence was minimal, and the organic matter inputs were largely biogenic.
- 1960s–1990s: Post-independence economic development and population growth drove a distinct shift in organic matter deposition trends, marked by rising pyrogenic PAH inputs that reflect increased fossil fuel combustion. There was also an increase in petroleum-derived n-alkanes from expanding transportation and industrial activities in the catchment. The decreasing CPI values and changes in n-alkane distributions indicate growing anthropogenic influence.
- Post-1990s: Urbanization has continued into the post-1990s period, as reflected in the most recent sediments obtained for this study. This period exhibits the highest concentrations of total PAHs and n-alkanes. The biomarker trends indicate evidence of mixed pyrogenic and petrogenic sources, with growing contributions from fossil fuel combustion. The variability in organic matter inputs reflects shifting energy and land use patterns.

## 5. Conclusions

There is a distinct change in BC, PAH, and n-alkane trends in the centennial-to-decadal record from sediment cores retrieved from both the Lake Victoria catchment and mid-lake sediments. The catchment sites, representing both rural and urban locations, have experienced a transition from biomass-burning signatures—primarily associated with natural forest fires and traditional agricultural practices in early history—to increased anthropogenic inputs from the mid-20th century onward. The rural sites typically indicate low concentrations of PAHs and n-alkanes that are dominated by long-chain compounds. Although these concentrations are still relatively low, these sites reflect a gradual transition from predominantly natural inputs to increasing anthropogenic contributions over the past century. The sediment cores from Homa Bay and Kisumu provide a valuable record of the transitioning urban centers in the Lake Victoria basin. They indicate higher concentrations of pyrolytic PAHs and short-chain n-alkanes and decreasing CPI and TAR values. This shift aligns with regional trends of post-independence development, rising population growth, and changes in land use patterns. The mid-lake is an integrated record of inputs from multiple sub-catchments, receiving organic matter through atmospheric deposition, riverine, and in-lake transport. The continually evolving depositional profiles at these sites highlight the complex interplay between human activities and the local environment, underscoring the significant impact of human-induced changes on the site’s environmental history. These findings highlight the cumulative effects of urbanization, industrial development, and land use changes on organic matter inputs to the Lake Victoria basin over the past century.

Management strategies should be tailored to address the specific challenges in both rural and urban settings. In urban areas, efforts should focus on reducing emissions from fossil fuel combustion, controlling industrial discharges, and managing urban runoff to mitigate anthropogenic pollution. Promoting sustainable agricultural practices, such as banning slash-and-burn techniques and curbing deforestation in rural areas, can help preserve the natural balance of organic matter inputs. Overall, preserving Lake Victoria’s ecological integrity requires integrated management approaches that consider both nutrient inputs and organic matter pollution from diverse sources across the basin. The continued monitoring and assessment of organic matter inputs are essential for informing policy decisions and implementing practical conservation efforts.

Future investigations should focus on expanding spatial sampling coverage over the LVB and enhance risk assessments by quantifying potential human risks associated with contaminated sediments and the consumption of fish and other local produce that may bioaccumulate or biomagnify toxic pollutants through the food chain. Additionally, advanced analytical techniques, such as compound-specific stable isotope analysis and source apportionment modeling using positive matrix factorization, could refine pollutant sources, transport pathways, and spatial distribution.

## Figures and Tables

**Table 2:** Summary of key ratios used to analyze PAH sources in sedimentary environments.

Ratio	Background	Interpretations	Reference
ΣLow − Molecular Weight PAHs/ ΣHigh − Molecular Weight PAH	LMW PAHs with two or three fused aromatic rings HMW PAHs with five to six fused aromatic rings	<1 attributed to pyrogenic origin >1 attributed to petrogenic origin	[34,43,58,59]
FLA/(FLA + Pyr)[Flouranthene/(Fluoranthene + Pyrene)]	If PAHs are from petroleum or other combustion sources	Pyrolytic origin (>0.5) Petrogenic origin (<0.4)	[34,60,61]
IP/(IP + BghiP) [Indeno (*1,2,3-cd*) pyrene/Indeno[*1,2,3-cd*]pyrene + benzo[*ghi*]perylene)]	Identify if the PAH is from petroleum, coal combustion, or other combustion sources	<0.2—petroleum >0.5—wood, grass, and/or coal combustion Between 0.2 and 0.5—petroleum combustion	[60]

**Table 3:** Summary of key ratios used to assess n-alkane sources in catchment and lake sediments.

Ratio	Background	Interpretation	Reference
Low-Molecular-Weight n-Alkanes (C_10–23_)/High-Molecular-Weight n-Alkanes (C_24_–C_36_)	LMW alkanes are attributed to the abundance of natural or anthropogenic petroleum input HMW indicates an abundance of degraded oil or terrestrial plants	<1 indicates heavy and degraded oil and terrestrial plant origin >1 suggests a sign of fresh oil input, e.g., petrol, diesel	[35,44]
Terrigenous Aquatic Ratio (TAR) $=\left(\frac{\left(C_{27}+C_{29}+C_{31}\right)}{\left(C_{15}+C_{17}+C_{19}\right)}\right)$	TAR is used to identify the source of n-alkanes by calculating the abundance of C_27_, C_29_, and C_31_ n-alkanes (from terrestrial plants) in comparison to C_17_, C_19_, and C_21_ n-alkanes (from algal input)	>1 indicates land-based biogenic sources <1 indicates aquatic/biogenic source	[40]
Carbon Preference Index (CPI) $=\frac{1}{2}\left(\frac{\left(C_{25}+C_{27}+C_{29}+C_{31}+C_{33}\right)}{\left(C_{24}+C_{26}+C_{28}+C_{30}+C_{32}\right)}+\frac{\left(C_{25}+C_{27}+C_{29}+C_{31}+C_{33}\right)}{\left(C_{26}+C_{28}+C_{30}+C_{32}+C_{34}\right)}\right)$	CPI is used to identify n-alkane sources, where C_21_-C_25_ alkanes are attributed to emergent plants and C_27_-C_35_ n-alkanes are attributed to terrestrial plants	Value from 5 to 10 indicates terrestrial plant origin Value close to 1 indicates petrogenic inputs	[62]

## Data Availability

The original contributions presented in this study are included in the article/Supplementary Materials. Further inquiries can be directed to the corresponding author(s).

## Supplementary Materials

The following supporting information can be downloaded at: https://www.mdpi.com/article/10.3390/toxics13020130/s1, Table S1: Geochemical analyses of sediments from the Lake Victoria catchment; Figure S1: US EPA 16PAH standard (500 ppb) and the identification of different peaks and internal standards. The standard was run in the same manner as the samples and used to calculate the recovery during the extraction process. Detection and quantification limits were assessed based on the 5-point calibration curve and indicated below; Figure S2: Distribution of PAH in samples from Lake Victoria catchment; Figure S3: Identification of peaks in the S4006 multi-alkane standard (C14-32 alkanes).
