# Supplementary material for: Reconstructing Historical Land Use and Anthropogenic Inputs in Lake Victoria Basin: Insights from PAH and n-Alkane Trends
Source: Toxics. 2025 Feb 10;13(2):130. doi: 10.3390/toxics13020130 (PMC11860610; doi:10.3390/toxics13020130)

Supplementary data

Table S1. Geochemical analyses of sediments from the Lake Victoria catchment.

Core	Depth (cm)	Age	%TOC	%BC	Tot PAH (ng/g)	LMW/HMW PAH	FLA/ (FLA+Pyr)	IP/ (IP+BghiP)	cPAH (ng/g)	%2-ring	%3-ring	%4-ring	%5-ring	%6-ring	Tot Alk (ng/g)	LMW/HMW Alk	CPI	TAR
	3	2012	3,63	0,30	74,87	3,70	0,57	0,48	7,42	0,88	78,80	13,92	3,96	2,44	10197	0,79	2,82	6,10
	6	2005	3,64	0,42	56,80	1,29	0,53	0,42	14,04	1,16	57,12	25,05	10,84	5,83	10276	0,56	4,80	10,62
	12	1999	2,43	0,24	36,85	1,46	0,54	0,47	7,99	1,80	59,37	25,44	8,59	4,80	5504	0,75	4,86	7,39
	22	1980	1,70	0,14	30,50	1,66	0,56	0,49	4,64	2,16	60,58	27,46	5,83	3,97	3576	1,25	3,69	3,65
	26	1972	1,49	0,15	107	6,04	0,55	0,45	6,14	0,62	85,49	9,76	2,56	1,56	5030	1,18	3,13	3,62
	32	1961	1,35	0,43	63,26	1,16	0,55	0,51	6,43	1,03	53,19	41,13	2,86	1,79	3346	1,80	2,76	2,25
SPK (Busia)	42	1941	1,20	0,22	70,61	5,15	0,57	0,46	5,60	0,94	82,79	11,25	3,29	1,73	3281	1,70	2,07	2,36
	53	1921	0,78	0,11	73,19	15,5	0,62	0,52	1,83	0,89	93,13	3,08	1,76	1,15	4136	1,97	1,70	2,70
	68	1909	0,61	0,18	56,58	2,81	0,54	0,48	5,87	2,67	71,06	20,89	3,34	2,03	3233	2,56	1,33	1,12
	77	1896	0,60	0,11	16,18	1,33	0,56	0,50	4,53	9,27	47,77	23,90	12,03	7,03	3536	1,62	1,14	1,94
	Min		*0,60*	*0,11*	*16,18*	*1,16*	*0,53*	*0,42*	*1,83*	*0,62*	*47,77*	*3,08*	*1,76*	*1,15*	*3233*	*0,56*	*1,14*	*1,12*
	Max		*3,64*	*0,43*	*107*	*15,5*	*0,62*	*0,52*	*14,04*	*9,27*	*93,13*	*41,13*	*12,03*	*7,03*	*10276*	*2,56*	*4,86*	*10,62*
	Avg		*1,74*	*0,23*	*58,58*	*4,01*	*0,56*	*0,48*	*6,45*	*2,14*	*68,93*	*20,19*	*5,51*	*3,23*	*5211*	*1,42*	*2,83*	*4,18*
	22	2004	13,79	0,88	622	5,95	0,63	0,59	29,15	0,75	86,13	9,76	2,41	0,94	147252	0,19	6,99	43,14
	26	2000	8,56	0,86	82,12	1,13	0,66	0,69	27,91	1,50	52,24	15,73	28,12	2,42	57402	0,35	4,31	19,04
	28	1997	6,31	1,44	212	1,73	0,61	0,74	54,59	0,96	65,01	8,28	25,09	0,66	44206	0,35	4,59	21,30
	34	1990	6,47	1,32	352	1,04	0,60	0,65	130	0,50	53,19	11,10	34,37	0,84	51764	0,29	5,58	26,48
	44	1977	1,90	0,87	67,57	0,52	0,63	0,96	34,62	1,58	35,98	14,83	46,96	0,66	13982	0,41	3,71	18,74
	49	1969	2,27	0,49	168	0,68	0,71	0,65	74,94	0,57	44,67	11,97	42,27	0,51	16433	0,46	2,99	12,93
KK (Siaya)	59	1960	0,98	0,24	151	1,44	0,65	0,68	21,12	2,96	56,61	36,57	3,38	0,48	9804	0,53	2,39	6,26
	77	1940	1,57	0,10	42,09	2,24	0,59	0,92	6,00	7,49	61,67	26,45	3,73	0,67	16311	1,02	1,93	3,07
	89	1929	1,85	0,10	157	2,15	0,66	0,65	21,69	3,36	65,59	26,23	4,04	0,77	18582	0,65	2,17	6,53
	95	1923	1,09	0,14	170	1,86	0,69	0,60	25,95	3,62	62,42	28,09	4,57	1,29	24895	0,55	2,60	8,45
	Min		*0,98*	*0,10*	*42,09*	*0,52*	*0,59*	*0,59*	*6,00*	*0,50*	*35,98*	*8,28*	*2,41*	*0,48*	*9804*	*0,19*	*1,93*	*3,07*
	Max		*13,79*	*1,44*	*622*	*5,95*	*0,71*	*0,96*	*130*	*7,49*	*86,13*	*36,57*	*46,96*	*2,42*	*147252*	*1,02*	*6,99*	*43,14*
	Avg		*4,48*	*0,64*	*202*	*1,87*	*0,64*	*0,71*	*42,59*	*2,33*	*58,35*	*18,90*	*19,49*	*0,92*	*40063*	*0,48*	*3,73*	*16,60*
	32	1929	28,90		176	0,10	0,63	0,57	108	2,28	7,02	50,51	31,93	8,26	2747	10,53	1,42	2,76
	48	1894	26,40		318	0,43	0,54	0,51	102	1,28	28,90	45,17	21,22	3,43	41773	1,50	2,47	43,16
	56	1876	27,56		283	0,41	0,85	0,57	134	1,28	27,60	39,50	26,72	4,91	39827	0,82	2,57	17,62
	64	1858	31,49		289	0,74	0,84	0,54	110	1,65	40,77	21,36	32,08	4,13	109168	0,98	1,38	16,30
	72	1840	34,11		242	1,10	0,83	0,58	63,08	2,39	50,17	22,95	20,53	3,96	54683	0,79	1,66	4,97
	80	1820	35,06		309	0,61	0,55	0,57	116	1,13	36,88	25,91	32,78	3,30	71736	1,12	1,51	26,00
	96	1777	40,13		175	0,11	0,79	0,60	124	2,71	6,83	23,18	62,99	4,29	60953	0,53	2,27	9,96
	104	1756	33,43		157	0,06	0,72	0,68	127	2,15	3,15	15,20	76,92	2,57	63051	1,31	1,44	54,70
	112	1735	34,61		172	0,04	0,51	0,75	146	2,37	1,29	11,99	82,74	1,62	60573	0,52	1,88	32,75
KP1A (Kapsabet)	120	1714	37,39		17,42	0,31	0,51	1,00	2,72	19,26	4,56	60,58	11,04	4,56	35847	0,39	1,90	96,41
	128	1690	30,05		17,35	0,31	0,51	1,00	2,71	19,30	4,50	60,60	11,05	4,55	31093	0,34	2,24	46,27
	144	1636	32,01		407	0,05	0,56	0,11	321	0,97	3,51	6,54	76,96	12,02	77854	0,36	2,43	22,47
	152	1609	32,47		88,88	0,06	0,52	0,99	68,95	4,76	1,23	16,58	73,79	3,65	8467	3,04	4,21	9,35
	168	1555	40,30		374	0,02	0,53	0,61	345	1,34	0,76	5,24	91,23	1,42	49326	0,41	2,00	31,54
	184	1491	40,13		413	0,04	0,52	0,68	369	1,01	3,28	6,55	87,04	2,13	29078	0,41	2,11	48,53
	192	1455	34,05		647	0,02	0,50	0,73	593	0,54	0,99	8,00	88,15	2,32	64522	0,24	2,37	53,93
	200	1420	33,03		573	0,02	0,53	0,61	528	0,85	0,73	6,47	90,53	1,42	60281	0,14	2,65	21,35
	Min		*26,40*		*17,35*	*0,02*	*0,50*	*0,11*	*2,71*	*0,54*	*0,73*	*5,24*	*11,04*	*1,42*	*2747*	*0,14*	*1,38*	*2,76*
	Max		*40,30*		*647*	*1,10*	*0,85*	*1,00*	*593*	*19,30*	*50,17*	*60,60*	*91,23*	*12,02*	*109168*	*10,53*	*4,21*	*96,41*
	Avg		*33,60*		*274*	*0,26*	*0,61*	*0,65*	*192*	*3,84*	*13,07*	*25,08*	*53,98*	*4,03*	*50646*	*1,38*	*2,15*	*31,65*
	13	1960	6,08	0,58	21,12	1,64	0,52	1,00	5,01	63,01	1,22	12,04	21,88	1,85	710272	0,46	8,68	16,81
	21	1867	3,16	0,64	47,99	1,05	0,74	0,60	12,21	9,79	41,50	28,62	14,94	5,14	451673	1,75	5,40	2,97
	31	1739	3,67	0,37	45,83	1,02	0,73	0,61	12,49	9,79	40,65	30,06	14,71	4,80	222964	1,01	8,44	9,52
	41	1609	4,26	0,37	83,46	1,53	0,71	0,64	18,14	4,73	55,82	25,76	9,11	4,58	437237	0,98	12,13	9,44
DK (Kisumu)	51	1546	2,75	0,71	43,06	0,48	0,70	0,64	18,18	5,83	26,40	41,02	20,88	5,87	919776	0,70	9,55	8,96
	63	1483	2,09	0,65	14,27	0,74	0,52	0,70	3,20	5,18	37,35	36,39	13,68	7,40	278777	2,78	1,47	2,08
	73	1417	1,40	0,31	4,76	0,22	0,51	0,69	1,53	14,20	4,12	44,65	21,21	15,81	168771	7,49	0,40	0,34
	81	1379	1,45	0,39	20,37	1,56	0,55	0,67	2,04	3,37	57,62	30,14	4,89	3,99	381465	3,96	1,37	0,89
	91	1305	1,55	0,13	13,30	0,58	0,61	0,59	3,68	4,99	31,88	42,14	16,44	4,55	214816	17,29	0,64	0,11
	Min		*1,40*	*0,13*	*4,76*	*0,22*	*0,51*	*0,59*	*1,53*	*3,37*	*1,22*	*12,04*	*4,89*	*1,85*	*168771*	*0,46*	*0,40*	*0,11*
	Max		*6,08*	*0,71*	*83,46*	*1,64*	*0,74*	*1,00*	*18,18*	*63,01*	*57,62*	*44,65*	*21,88*	*15,81*	*919776*	*17,29*	*12,13*	*16,81*
	Avg		*2,93*	*0,46*	*32,68*	*0,98*	*0,62*	*0,68*	*8,50*	*13,43*	*32,95*	*32,31*	*15,30*	*6,00*	*420639*	*4,05*	*5,34*	*5,68*
	2		2,77	0,72	178	0,64	bdl	0,46	15,93	0,47	75,57	16,55	5,19	2,22	489150	0,58	0,34	2,29
	8		2,65	0,39	23,57	1,68	0,58	0,49	3,59	5,65	57,05	21,58	10,34	5,38	72884	1,64	1,96	2,93
	20		2,48	0,45	216	3,38	0,56	0,65	12,87	0,87	76,28	21,13	0,79	0,93	306869	0,66	2,65	5,52
	40		2,04	1,15	686	0,58	0,55	0,55	198	0,12	36,71	58,03	4,65	0,48	24591	0,69	2,32	6,26
	48		2,35	1,17	263	2,17	0,57	0,55	24,29	0,28	68,15	29,68	1,36	0,52	472940	0,73	11,35	17,53
	60		3,33	0,66	76,62	1,77	0,73	0,34	8,90	2,96	60,99	21,78	5,55	8,73	366679	0,69	4,73	5,92
HK (Homa Bay)	64		2,82	0,73	317	2,04	0,56	0,77	48,62	0,23	66,92	24,11	7,24	1,50	295055	0,64	5,31	6,22
	72		2,84	0,52	58,21	0,83	0,61	0,70	22,51	4,18	41,13	32,82	14,97	6,90	218519	0,98	3,28	4,93
	80		3,07	0,71	367	0,83	0,67	0,58	124	1,03	44,28	40,32	11,60	2,77	35847	0,55	2,57	6,57
	87		2,63	0,53	244	1,13	0,73	0,63	58,47	2,16	50,87	38,01	7,24	1,72	413367	0,52	4,21	8,06
	96		1,66	0,37	693	0,75	0,58	0,56	161	0,26	42,55	47,49	8,38	1,32	185108	0,54	1,84	20,11
	Min		*1,66*	*0,37*	*23,57*	*0,58*	*0,55*	*0,34*	*3,59*	*0,12*	*36,71*	*16,55*	*0,79*	*0,48*	*24591*	*0,52*	*0,34*	*2,29*
	Max		*3,33*	*1,17*	*693*	*3,38*	*0,73*	*0,77*	*198*	*5,65*	*76,28*	*58,03*	*14,97*	*8,73*	*489150*	*1,64*	*11,35*	*20,11*
	Avg		*2,60*	*0,67*	*284*	*1,44*	*0,61*	*0,57*	*61,70*	*1,66*	*56,41*	*31,95*	*7,03*	*2,95*	*261910*	*0,75*	*3,69*	*7,85*
	1	1996			203	4,81	bdl	0,75	30,09	10,52	72,26	0,00	7,69	9,52	209	0,44	4,38	15,93
	11	1970			147	1,67	0,57	0,73	23,23	4,69	57,89	22,63	10,19	4,60	33,74	0,65	4,88	7,95
	20	1945	19,28	2,41	123	0,49	1,00	0,57	50,28	5,45	27,40	17,46	29,34	20,36	95,06	0,78	3,68	5,94
	29	1924	20,06	3,02	119	1,38	1,00	0,77	51,03	22,28	18,47	10,52	27,85	20,88	21,53	0,80	2,39	5,60
	40	1880			148	0,40	1,00	0,66	87,67	4,60	24,22	9,82	54,15	7,21	47,10	0,48	4,82	9,68
	41				128	0,29	1,00	0,64	77,80	14,63	7,57	3,19	33,75	40,87	355	0,47	3,75	14,33
	42		20,33	2,65	1935	0,97	1,00	0,69	123	8,21	77,46	8,25	3,20	2,88	448	0,64	4,01	8,98
	49	1850	20,55	3,32	207	0,24	0,82	0,66	108	8,92	10,38	24,37	34,17	22,16	42,75	0,42	1,79	8,21
LV95 (Lake Victoria)	60	1807	19,11	2,35	90,17	6,94	1,00	0,95	25,83	28,05	18,48	18,71	19,18	15,59	155	0,39	5,01	17,82
	72	1777	19,11	6,15	968	0,60	0,61	0,63	41,02	10,09	77,32	8,65	2,19	1,75	279	0,74	3,42	8,16
	79				28,65	5,98	1,00	0,68	16,47	23,49	15,07	0,00	32,76	28,67	271	0,36	4,51	18,79
	80	1734	19,62	2,73	371	0,07	0,11	0,72	45,66	7,94	67,81	9,23	8,08	6,95	184	0,49	4,95	12,88
	90	1704			1040	11,35	1,00	0,91	450	2,06	35,56	19,59	39,85	2,93	54,87	1,72	4,96	2,71
	Min		*19.11*	*2,35*	*28,65*	*0,24*	*0,57*	*0,57*	*16,47*	*4,60*	*7,57*	*0,00*	*2,19*	*1,75*	*21,53*	*0,36*	*1,79*	*5,60*
	Max		*20.55*	*6,15*	*1935*	*6,94*	*1,00*	*0,95*	*123*	*28,05*	*77,46*	*24,37*	*54,15*	*40,87*	*448*	*0,80*	*5,01*	*18,79*
	Avg		*19,72*	*3,23*	*424*	*1,90*	*0,90*	*0,70*	*60,49*	*13,04*	*33,43*	*12,36*	*24,68*	*16,50*	*175*	*0,57*	*3,83*	*10,55*

Note: bdl - below detection limit of one of the compounds

Figure S1. US EPA 16PAH standard (500 ppb) and the identification of different peaks and internal standards. The standard was run in the same manner as the samples and used to calculate the recovery during the extraction process. Detection and quantification limits were assessed based on the 5-point calibration curve and indicated below.

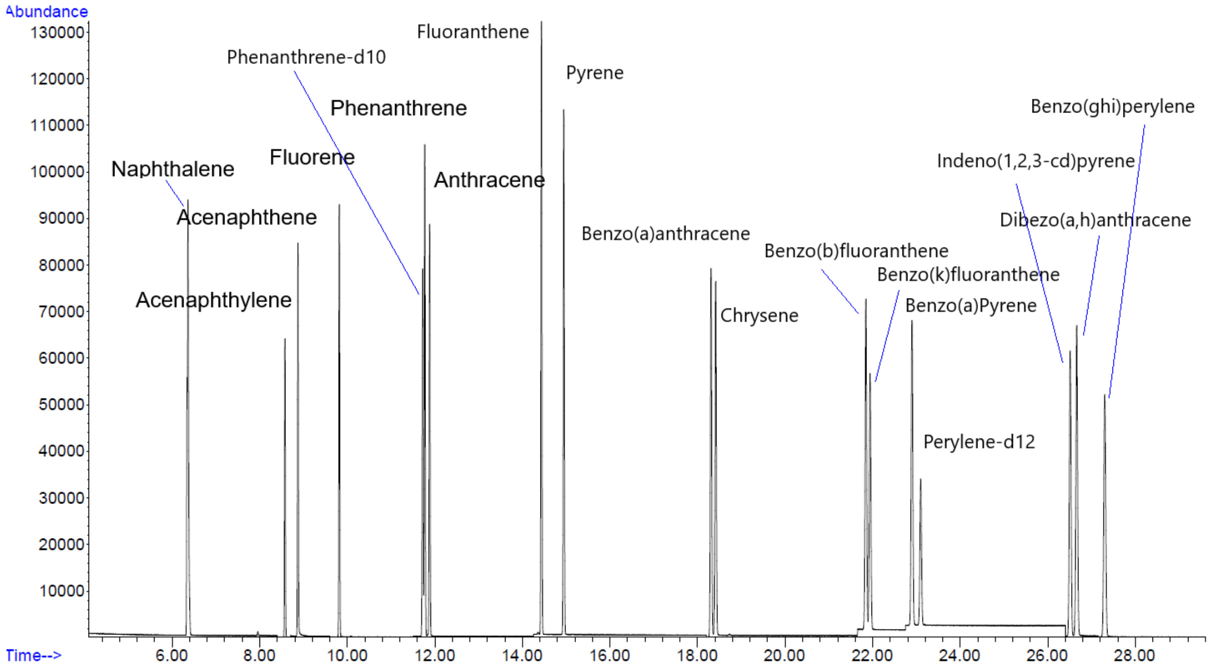

ppb	LOD	LOQ
Naphthalene	67	205
Acenaphthylene	195	593
Acenaphthene	163	494
Fluorene	127	387
Phenanthrene	102	311
Anthracene	122	371
Fluoranthene	51	154
Pyrene	77	234
Benzo(a)anthracene	147	446
Chrysene	107	325
Benzo(b)fluoranthene	63	192
Benzo(k)fluoranthene	63	192
Benzo(a)Pyrene	51	157
Indeno(1,2,3-cd)pyrene	56	172
Dibezo(a,h)anthracene	39	120
Benzo(ghi)perylene	40	121

LOD = 3.3 * y-intercept/slope

LOQ = 10 * y-intercept/slope

Figure S2. Distribution of PAH in samples from Lake Victoria catchment.

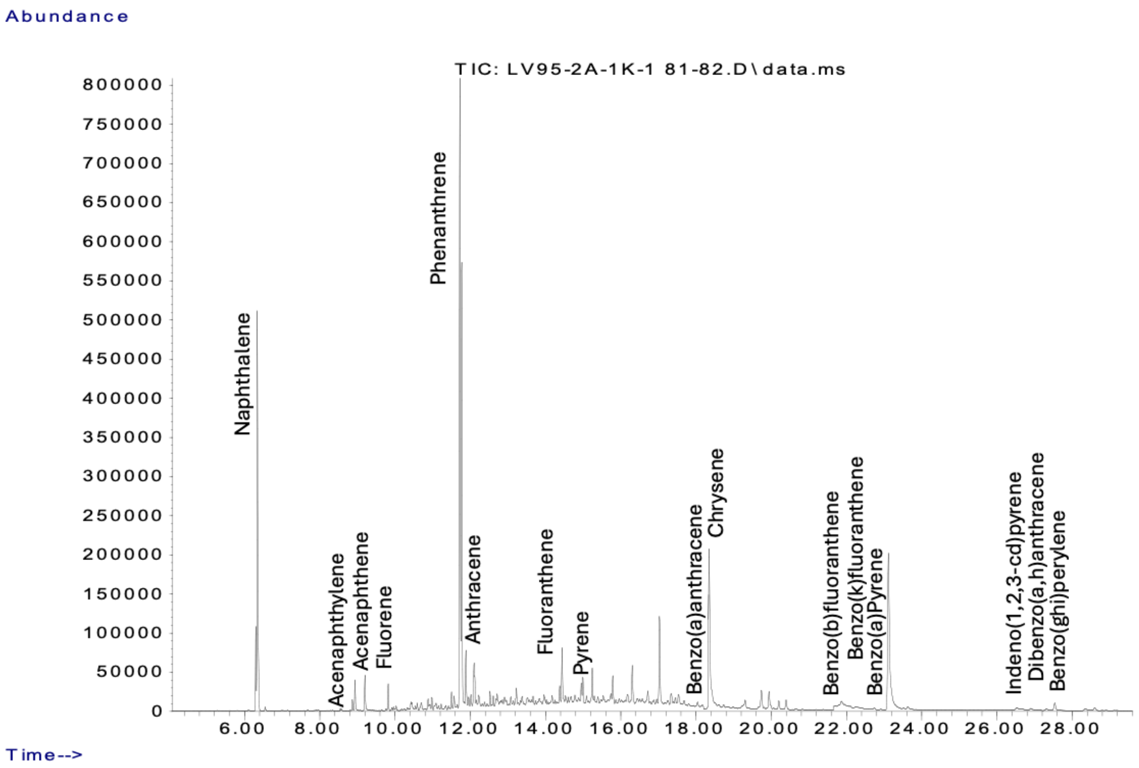

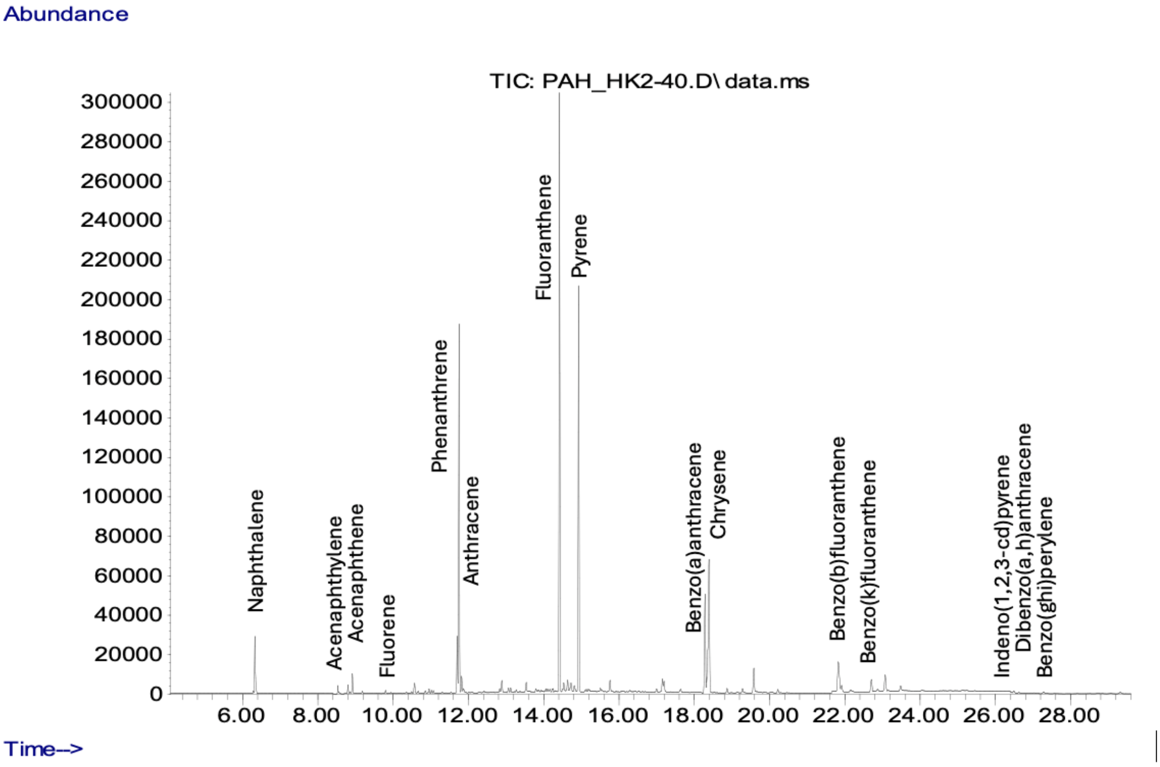

Figure S3. Identification of peaks in the S4006 multi-alkane standard (C_{14-32} alkanes).

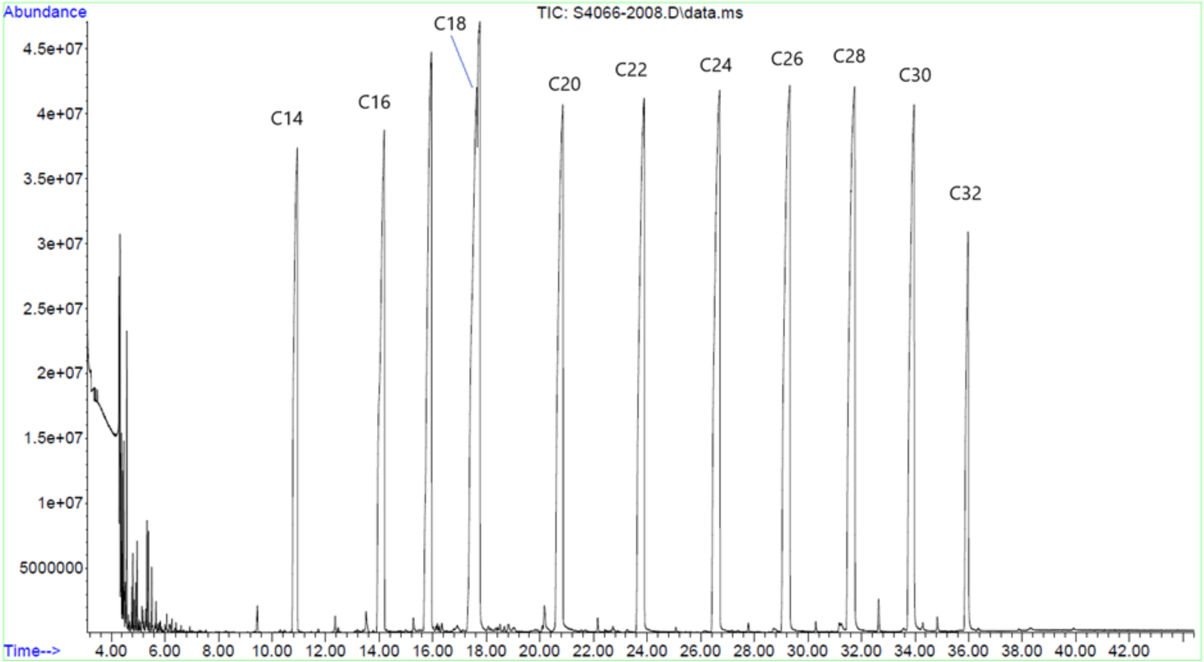

Supplement: Supplementary file 1 [file toxics-13-00130-s001.zip › toxics-3404347-supplementary.pdf]
